# Statistical modeling for Ree-Eyring nanofluid flow in a conical gap between porous rotating surfaces with entropy generation and Hall Effect

**DOI:** 10.1038/s41598-022-25136-y

**Published:** 2022-12-07

**Authors:** Muhammad Rooman, Anum Shafiq, Zahir Shah, Narcisa Vrinceanu, Wejdan Deebani, Meshal Shutaywi

**Affiliations:** 1 Department of Mathematical Sciences, University of Lakki Marwat, Lakki Marwat, 28420 Khyber Pakhtunkhwa Pakistan; 2 School of Mathematics and Statistics, Nanjing University of Information Science and Technology, Nanjing, 210044 China; 3 Department of Industrial Machines and Equipments, Faculty of Engineering, “Lucian Blaga” University of Sibiu, 10 Victoriei Boulevard, 5500204 Sibiu, Romania; 4 Department of Mathematics, College of Science and Arts, King Abdul-Aziz University, Rabigh, Saudi Arabia

**Keywords:** Engineering, Mathematics and computing, Physics

## Abstract

The attention of the current study is on the flow of a non-Newtonian incompressible Cu-Water nanofluid flow. The water is assumed as base fluid, while copper is used as nanoparticles. The Ree-Eyring prototype describes the performance of non-Newtonian nanofluids. There is a conical gap that nanofluid flow fills among the plane disc and the cone's stationary/rotational porous faces. Additionally taken into account are heat, mass transfer, and entropy production. The given mathematical model is unique due to the effects of a vertically applied Hall Effect, Ohmic dissipation, viscous dissipation, and chemical processes. The Ree-Eyring fluid constitutive equations, as well as the cylindrical coordinates, have been interpreted. The model equations for motion, heat, and concentration can be changed in the collection of non-linear ODEs by employing the applicable similarity transform. This method allocates a couple of nonlinear ODEs relating to velocity, temperature, and concentration distributions. The shooting scheme (bvp4c technique) is used to solve these equations numerically. Statistical analysis like probable error, correlation, and regression are exploited. The probable error is estimated to compute the consistency of the calculated correlation features. The theoretical data is analyzed in both graphical and tabular forms. The modeled parameters like, magnetic number, porosity parameter, Eckert number, chemical reaction parameter, Brownian motion parameter, thermophoretic parameter, Schmidt number, Hall recent parameter, radiation parameter, and volume fraction are discussed in details graphically and theoretically. The outcomes indicate that the velocity components are greater for greater values of nanoparticle volume fraction and Weissenberg number, whereas for enormous values of magnetic and porosity parameters, the velocity components fall.

## Introduction

Many studies on the flow of nanofluid and heat transmission with water as a base fluid have been conducted over the last few decades. Choi and Eastman^1^ first used the term “nanofluids” in 1995. They were the first to increase heat conductivity by adding nanoscale metal, oxide, and carbide particles to base fluids. Quite a few metal and metal oxide nanoparticles were tested by Yu et al.^2^ in a variety of base fluids, and the findings were encouraging, but there are still many unanswered problems regarding this interruption of nanostructured materials. In comparison to foundation fluids like water, oil, and ethylene glycol, their study aims to increase thermophysical qualities including thermal conductance, viscosity, thermal viscosity, and turbulent heat transmission coefficients. Only one or two of the suggested heat transmission frameworks are included in a significant portion of nanofluid simulations. Analysis of the heat transmission coefficients of nanofluids in ordinary convection or the effectual thermal diffusivity of nanofluids has not established much elementary investigation; for illustration, see Wong et al.^3^ Shah et al.^4^ studied the involvement of titanium dioxide droplets in ecosystems. Khan et al.^5^ probed the role of slide consequence in Eyring-Powell liquid heat transfer with grapheme particles. Under the heading of time-dependent magnetic fields, the idea of the thin film is additionally examined. The homotopic solution strategy was used to locate analytical answers to the flow-governing equations. When compared to the Prandtl number, they saw a drop in the temperature field. Alobaid et al.^6^ investigate the effects of carbon-based nanomaterials to observe the characteristics of damaged soils through testing. Ali et al.^7^ established the significance of thermal emission and heat genesis in the stagnation point flow of a viscous liquid passing through a straight cylinder by integrating thermophoresis and Brownian motion. They were successful in solving combined nonlinear ordinary differential equations (ODEs) in an unbounded domain using the shooting method. Along with the loss in temperature about the Prandtl number and the radiation parameter, they also noted the decrease in velocity in proportion to the mounting curvature parameter values. Non-Newtonian liquids outperform viscous fluids in an extensive variety of industrialized and engineering fields. The viscosity and behavior of other fluids are different from the non-Newtonian fluids, such as molasses, silicon oils, polymer solutions, gypsum paste, and so on. Due to this viscosity difference, they are extensively used in industries. Additionally, it is challenging to elaborate on the behavior of all of these liquids using a lone simple equation. As a consequence, so many categories are developed to illustrate the behaviors of non-Newtonian liquids. Many of these researchers claim that the Newtonian model for greater and lower shear rates may be derived from the Ree-Eyring fluid flow model, which is the more substantial model^8,9^. The influence of heat transmission on the contractility flow of Ree-Eyring fluids within a revolving surround was investigated by Hayat et al.^10^. This study shows that the rotation parameter has a significant impact on fundamental and incidental velocities. Additionally, it demonstrates that a non-uniform heat source causes the heat transfer coefficient to rise when it is present in the heat equation. Hayat et al.^11^ investigated Ree-Eyring nanofluids as well as Arrhenius mobilization power and entropy minimization in the middle of two stretchy rotating discs. The nonlinear system is transformed through appropriate transfigurations and analytically worked out by OHAM. Tanveer and Malik^12^ looked into the thermal efficiency of Ree-Eyring nanofluid peristaltic flow.

The Arrhenius instigation energy is essential for a chemical reaction to begin, and it's the least significant. In 1889, Arrhenius devised the term. Activation energy acts as a barrier between unreacted particles or bits. Once this barrier is overcome, a reaction takes place chemically, and the atoms or molecules with more energy than the fence will traverse the blockade. Awad et al.^13^ used the Spectral Relaxation Process to examine how actuation energy and binary chemical processes affect a viscous rotational motion. It is found that increasing the dimensionless actuation energy values widens the concentration distribution. Also noted is the monotonic degradation of the velocity field concerning a low fluid spinning level. Lu et al.^14^ estimated a 3D nano liquid flow via a movable, expanded plate using the simulated effects of Arrhenius actuation energy with dualistic chemical reaction. The suggested computational model takes into account anisotropic slide at the boundary, thermal radiation, and gyrotactic microorganisms. It is shown that, in comparison to the non-dimensional activation energy, the confined density of microorganisms drops. Khan et al.^15^ examined numerically nanofluid flow using second-grade model. They studied the effect of Arrhenius actuation energy with chemical action in a flexible media. The flow analysis also considers the generation of entropy and thermal radiation. The concentration is claimed to decrease for high chemical reaction and actuation parameter values.

Today, applications of magnetohydrodynamics with strong applied magnetic fields are chosen to be used to research the effects of magnetohydrodynamics. This shows the importance of Hall and Ion slip, and their substantial outcome on the magnitude, course of present density, and course of the term of magnetic force^16^. When exposed to a magnetic field, ionized fluids behave considerably differently from non-ionized fluids. The three (3) primary effects of ionized fluids are the magnetic force produced by a functional magnetic field, the Hall force produced by electron impacts, and the ion slip force produced by ion collisions. The majority of published publications discuss the effects of the Lorentz force on flows. There is a possibility of less research under Hall's influence. However, there is a scarcity of works that highlight the effects of either Ion or hall slip. Tangent hyperbolic nanofluid was studied by Abdelsalam and Batti^17^ under the influence of an ion Hall slide and a non-uniform channel caused by a chemical reaction. The study's key conclusions were that coupled solutions for absorption profiles provided by a chemical reaction allow for a more precise assessment of the Brownian motion parameter. Aluminum, silver, and copper oxides are among the metallic units in the nanofluid flow with their oxides, which Nawaz et al.^18^ believe are submerged in the blood with ion and Hall skid repercussions along with heat production and fascinations. By using Ion and Hall glide effects, a significant drop in the heat decadence in the apparent magnetic field is discovered.

One of the most important aspects of fluid mechanics is entropy generation analysis. Thermal device performance is directly dependent on the accessible quantity of work, which reduces due to flow unalterable and causes supplementary disarray. To maximize the thermal competency of appliances, it is necessary to examine the dynamics of entropy creation. The results of a real magnetic field passing through three spherical cylinders, each with porous hollow and wavy walls, were studied. Dogonchi et al.^19^ examined the entropy production conduct in natural transmission hybrid nanofluid rheology. Sahoo et al.^20^ investigated entropy generation with destructive heat transport in various transmissions MHD Casson nanofluid dynamics underneath the influence of Hall present and thermal radiation. The findings show that, for the concentration ratio (:) parameter, Brinkman number, and diffusive variable, entropy production increases significantly whereas Bejan number falls for all of these parameters. Ahmad et al.^21^ probed the flow of viscous nanoparticles in five (5) distinct shapes as a function of time. Rehman et al.^22^ probed the thermal behavior of a revolving nanofluid with entropy formation. Alsarraf et al.^23^ used a two-phase composition model to study the flow of nanofluid with various forms of nanoparticles in a mini-channel heat exchanger. To cool a supercomputer circuit board, Moradikazerouni et al.^24^ investigate the outcome of five alternative micro-channel heat sink channel shapes under forced deportation. By taking into consideration the fact that certain nanofluids may act on the basis of non-Newtonian power law fluids with the power law index close to unity, Jafarimoghaddam et al.^25^ discovered significant relationships for estimating skin friction coefficient and convective heat transfer coefficient arise respecting the classical Blasius flow. A substantial amount of work was nearly concluded^26,27^.

The foregoing investigations show that no attempt has been made to date to analyze the entropy production of the three-dimensional nanofluid using Ree-Eyring model around the disc and cone by way of operating or static in a porous media with magnetic field effect. This study investigates the outcome of copper nanoparticles on the thermophysical characteristics of water. It has numerous uses in both science and technology. The flow equations are transformed into regular systems, and the shooting scheme is used to handle them (bvp4c). The impacts of physical factors on velocity, temperature, concentration, entropy formation, and Bejan number are shown in the figures. Intriguing physical entities are compared to the numerical results for surface drag force, temperature gradient, and Sherwood number. The existing work's originality is highlighted.

- The three-dimensional Ree-Eyring nanofluid flow is considered in this paper.
- In the present case, the magnetic field and porous medium are applied vertically to the flow pattern.
- The Shooting technique (bvp4c) package was used to resolve the nonlinear problem.
- The entropy production and Bejan number are also considered in this article.

## Mathematical formulation and geometry

**Figure 1:**
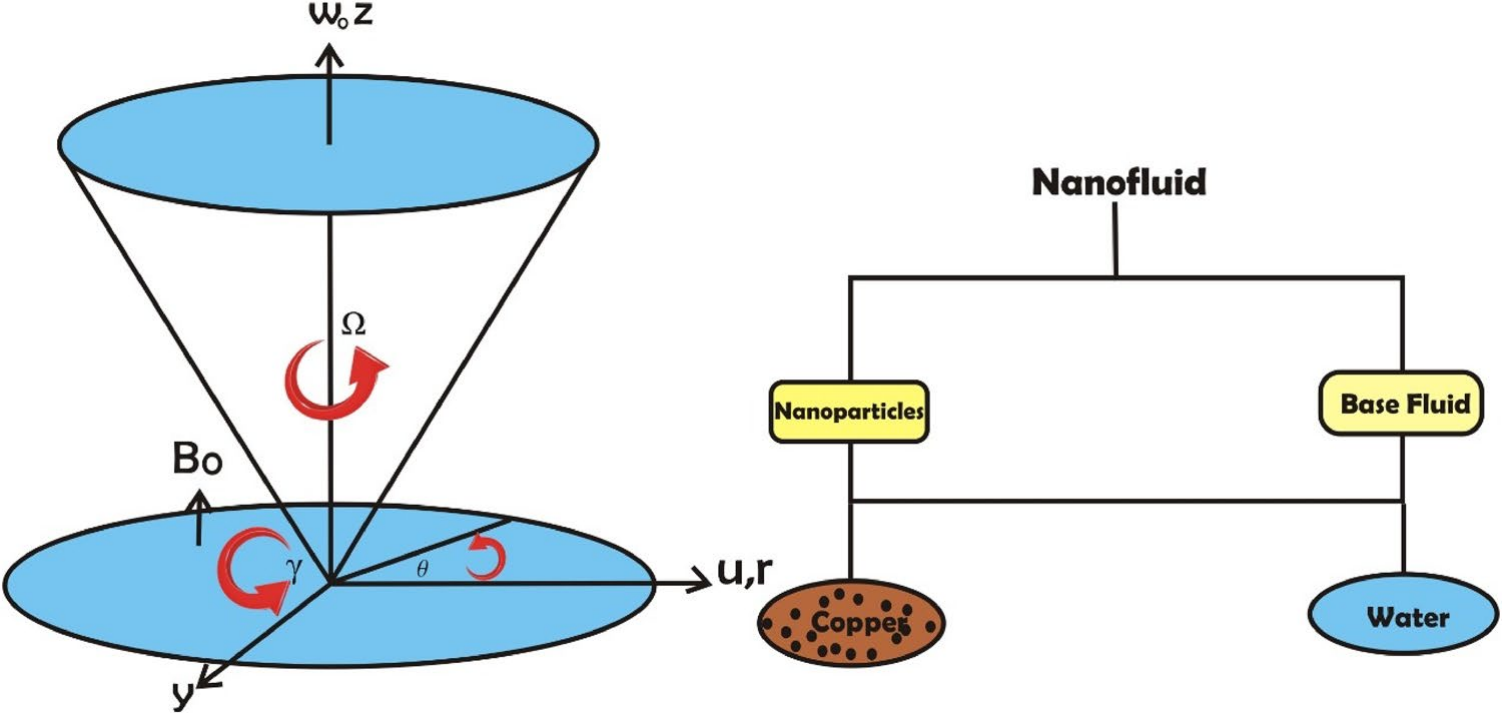
Flow chart of the flow model.

Consider the nanofluid flow amongst a conical and a disc that is incompressible, stable, axisymmetric, hydrodynamic, and influenced by the Hall Effect and a porous media. In the cylindrical coordinate $\left(r, \theta , z\right)$, both equipment (disc & cone) are presumed to be stationary or revolving. The $\Omega$ and $\omega$ indicate the angular velocities of the cone and disc, respectively. The magnetic field's strength along the z-axis is measured as, ${B}_{0}$. Figure 1 depicts the flow mechanism. Additionally, thermal radiation, viscous dissipation, and Ohmic dissipation are used to investigate heat transport, and the concentration of nanoparticles as a result of chemical reactions is determined. Effective application of the phenomena has been made to a disc's surface with radically changing wall temperature ${T}_{w}={T}_{\infty }+c{r}^{n}$, where $c$ and $n$ are held constant and temperature of cone wall is ${T}_{\infty }$. The pressure p within the conical gap depends on both the axial $z$ and circular $r$ dircection.

### Governing equations of fluid flow motion and heat transfer with and appropriate boundary circumstances

The Ree-Eyring fluid model's extra stress tensor is ^11^:

1 $$ {\varvec{S}}_{{{\varvec{ij}}}} = \left[ {\mu_{nf} + \frac{1}{B\lambda }{\text{Sin}} h^{ - 1} \left( {\frac{\lambda }{{c_{1} }}} \right)} \right]{\varvec{A}}_{1} $$

where

2 $$ \lambda = \sqrt {\frac{1}{2}tr\left( {{\varvec{A}}_{1} } \right)^{2} } $$

since

3 $$ {\text{Sin}} h^{ - 1} \left( {\frac{\lambda }{{c_{1} }}} \right) \approx \frac{\lambda }{{c_{1} }} \;for\; \frac{\lambda }{{c_{1} }} \le 1 $$

so

4 $$ {\varvec{S}}_{{{\varvec{ij}}}} = \left( {\mu_{nf} + \frac{1}{{Bc_{1} }}} \right){\varvec{A}}_{1} $$

where $B, {c}_{1}$ are meterial parameters, ${\mu }_{nf}$ is dynamic viscosity of nanofluid, and first Revlian Ericksen tensor is denoted by ${{\varvec{A}}}_{1}$ which are articulated as^11^:

5 $$ {\varvec{A}}_{1} = grad\left( {\varvec{V}} \right) + \left( {grad\left( {\varvec{V}} \right)} \right)^{T} $$

On the basis of the preceding assumption, the governing equations are as follows^20,22,26^:

6 $$ \frac{\partial u}{{\partial r}} + \frac{u}{r} + \frac{\partial w}{{\partial z}} = 0 $$

7 $$ \begin{array}{*{20}c} {\rho_{nf} \left( {u\frac{\partial u}{{\partial r}} + w\frac{\partial u}{{\partial z}} - \frac{{v^{2} }}{r}} \right) = \left( {\mu_{nf} + \frac{1}{{Bc_{1} }}} \right)\left( {2\frac{{\partial^{2} u}}{{\partial r^{2} }} + \frac{{\partial^{2} u}}{{\partial z^{2} }} + \frac{{\partial^{2} w}}{\partial r\partial z} + \frac{2}{r}\frac{\partial u}{{\partial r}} - \frac{2u}{{r^{2} }}} \right)} \\ \quad\quad{ - \frac{{\sigma_{nf} }}{{\rho_{nf} }}\frac{{B_{0}^{2} }}{{1 + m^{2} }}\left( {u - mv} \right) - \frac{{\mu_{nf} }}{K}u} \\ \end{array} $$

8 $$ \begin{array}{*{20}c} {\rho_{nf} \left( {u\frac{\partial v}{{\partial r}} + w\frac{\partial v}{{\partial z}} + \frac{uv}{r}} \right) = \left( {\mu_{nf} + \frac{1}{{Bc_{1} }}} \right)\left( {\frac{{\partial^{2} v}}{{\partial r^{2} }} + \frac{{\partial^{2} v}}{{\partial z^{2} }} + \frac{1}{r}\frac{\partial v}{{\partial r}} - \frac{v}{{r^{2} }}} \right)} \\\qquad\qquad\;\; { - \frac{{\sigma_{nf} }}{{\rho_{nf} }}\frac{{B_{0}^{2} }}{{1 + m^{2} }}\left( {v - mu} \right) - \frac{{\mu_{nf} }}{K}v} \\ \end{array} $$

9 $$ \rho_{nf} \left( {u\frac{\partial w}{{\partial r}} + w\frac{\partial w}{{\partial z}}} \right) = \left( {\mu_{nf} + \frac{1}{{Bc_{1} }}} \right)\left( {\frac{{\partial^{2} w}}{{\partial r^{2} }} + \frac{{\partial^{2} w}}{{\partial z^{2} }} + \frac{1}{r}\frac{\partial w}{{\partial r}}} \right) $$

10 $$ \begin{aligned} \left( {\rho C_{p} } \right)_{nf} \left( {u\frac{\partial T}{{\partial r}} + w\frac{\partial T}{{\partial z}}} \right) = & k_{nf} \left( {\frac{{\partial^{2} T}}{{\partial r^{2} }} + \frac{{\partial^{2} T}}{{\partial z^{2} }} + \frac{1}{r}\frac{\partial T}{{\partial r}}} \right) - \frac{{\partial q_{r} }}{\partial z} + \sigma_{nf} B_{0}^{2} \left( {u^{2} + v^{2} } \right) \\ & + \tau \left[ {D_{B} \left( {\frac{\partial T}{{\partial Z}}\frac{\partial C}{{\partial Z}} + \frac{\partial T}{{\partial r}}\frac{\partial C}{{\partial r}}} \right) + \frac{{D_{T} }}{{T_{\infty } }}\left( {\left( {\frac{\partial T}{{\partial r}}} \right)^{2} + \left( {\frac{\partial T}{{\partial Z}}} \right)^{2} } \right)} \right] \\ & + \left( {\mu_{nf} + \frac{1}{{Bc_{1} }}} \right)\left[ {2\left( {\frac{\partial u}{{\partial r}}} \right)^{2} + 2\left( {\frac{\partial w}{{\partial z}}} \right)^{2} + \left( {\frac{\partial v}{{\partial z}}} \right)^{2} + 2\left( \frac{u}{r} \right)^{2} + \left( {\frac{\partial u}{{\partial z}} + \frac{\partial w}{{\partial r}}} \right)^{2} + \left( {\frac{\partial v}{{\partial r}} - \frac{v}{r}} \right)^{2} } \right] \\ \end{aligned} $$

11 $$ u\frac{\partial C}{{\partial r}} + w\frac{\partial C}{{\partial z}} = D_{B} \left( {\frac{{\partial^{2} C}}{{\partial r^{2} }} + \frac{{\partial^{2} C}}{{\partial z^{2} }} + \frac{1}{r}\frac{\partial C}{{\partial r}}} \right) + \frac{{D_{T} }}{{T_{\infty } }}\left( {\frac{{\partial^{2} T}}{{\partial r^{2} }} + \frac{{\partial^{2} T}}{{\partial z^{2} }} + \frac{1}{r}\frac{\partial T}{{\partial r}}} \right) - R_{1} \left( {C - C_{\infty } } \right) $$

The following is a way to express the boundary conditions:

12 $$ \begin{array}{*{20}c} {u = U_{w} , v = r\omega , w = 0, T = T_{w} , C = C_{w} \;at \; z = 0} \\ {u = 0, v = r\Omega , w = 0, T = T_{\infty } , C = C_{\infty } \; at\; z = r\tan \gamma } \\ \end{array} $$

### Considered nanofluid assets

The nanofluid representations are given by^1–5,26^ and physical features are specified in Table 1.

13 $$ \left. {\begin{array}{*{20}c} {\begin{array}{*{20}c} {\mu_{nf} = \mu_{f} \left( {1 - \phi } \right)^{ - 2.5} } \\ {\rho_{nf} = \rho_{f} \left( {1 - \phi } \right) + \phi \rho_{s} } \\ {\left( {\rho c_{p} } \right)_{nf} = \left( {\rho c_{p} } \right)_{f} \left( {1 - \phi } \right) + \phi \left( {\rho c_{p} } \right)_{s} } \\ \end{array} } \\ {k_{nf} = k_{f} \left[ {\frac{{k_{s} + 2k_{f} - 2\phi \left( {k_{f} - k_{s} } \right)}}{{k_{s} + 2k_{f} + 2\phi \left( {k_{f} - k_{s} } \right)}}} \right]} \\ \end{array} } \right\} $$

**Table 1:** Some physical features of Copper & water^1^.

Nanofluid	$\rho \left(kg/{m}^{3}\right)$	${C}_{p}\left(1/kg k\right)$	$k \left(w/mk\right)$
Water	997.1	4179	0.613
Copper	385	8933	400

### Applicable similarity transformations and modeled ODE’S

The controlling non-linear PDE’s are transformed into ODE’s by a sufficient correspondence inversion. The following similarity transformations are pertinent:

14 $$ \begin{array}{*{20}c} {u = \frac{{\upsilon_{f} }}{r}F\left( \eta \right) = U_{w} \left( \eta \right), v = \frac{{\upsilon_{f} }}{r}G\left( \eta \right), w = \frac{{\upsilon_{f} }}{r}H\left( \eta \right)} \\ {\theta \left( \eta \right) = \frac{{T - T_{\infty } }}{{T_{w} - T_{\infty } }}, \chi \left( \eta \right) = \frac{{C - C_{\infty } }}{{C_{w} - C_{\infty } }}, \;and\; \eta = \frac{z}{r}} \\ \end{array} $$

By methodically applying these changes to the earlier leading equations of motion, one can learn more.

15 $$ H^{\prime} - \eta F^{\prime} = 0, $$

16 $$ \begin{array}{*{20}c} {2\left( {1 + \left( {1 - \phi } \right)^{2.5} We} \right)\left( {\left( {1 + \eta^{2} } \right)F^{\prime\prime} + 3\eta F^{\prime}} \right) - \frac{{\sigma_{nf} }}{{\sigma_{f} }}\left( {1 - \phi } \right)^{2.5} \frac{M}{{1 + m^{2} }}\left( {F - mG} \right)} \\ { - \left( {1 - \phi } \right)^{2.5} \left( {1 - \phi + \phi \frac{{\rho_{s} }}{{\rho_{f} }}} \right)\left( {F^{2} + \eta F^{\prime}F + G^{2} - HF^{\prime}} \right) - \beta F = 0} \\ \end{array} $$

17 $$ \begin{array}{*{20}c} {\left( {1 + \left( {1 - \phi } \right)^{2.5} We} \right)\left( {\left( {1 + \eta^{2} } \right)G^{\prime\prime} + 3\eta G^{\prime}} \right) - \frac{{\sigma_{nf} }}{{\sigma_{f} }}\left( {1 - \phi } \right)^{2.5} \frac{M}{{1 + m^{2} }}\left( {G - mF} \right)} \\ { - \left( {1 - \phi } \right)^{2.5} \left( {1 - \phi + \phi \frac{{\rho_{s} }}{{\rho_{f} }}} \right)\left( {HG^{\prime} - \eta FG^{\prime}} \right) - \beta G = 0} \\ \end{array} $$

18 $$ \begin{array}{*{20}c} {\left( {1 + \left( {1 - \phi } \right)^{2.5} We} \right)\left( {\left( {1 + \eta^{2} } \right)H^{\prime\prime} + 3\eta H^{\prime} + H} \right) - } \\ {\left( {1 - \phi } \right)^{2.5} \left( {1 - \phi + \phi \frac{{\rho_{s} }}{{\rho_{f} }}} \right)\left( {HH^{\prime} - \eta FH^{\prime} - FH} \right) = 0} \\ \end{array} $$

19 $$ \begin{gathered} \frac{{k_{nf} }}{{k_{f} }}\left\{ {\left( {\left( {1 + \eta^{2} } \right) + \frac{{k_{f} }}{{k_{nf} }}Rd} \right)\theta^{\prime\prime} + \eta \theta^{\prime}} \right\} + \left( {1 + \eta^{2} } \right)Pr\left( {Nb\theta^{\prime}\chi^{\prime} + Nt\theta^{{\prime}{2}} } \right) \hfill \\ - Pr\left( {1 - \phi + \phi \frac{{\left( {\rho C_{p} } \right)_{s} }}{{\left( {\rho C_{p} } \right)_{f} }}} \right)\left( {H - \eta F} \right)\theta^{\prime} + \frac{{\sigma_{nf} }}{{\sigma_{f} }}MPrEc\left( {F^{2} + G^{2} } \right) \hfill \\ + \frac{1}{{\left( {1 - \phi } \right)^{2.5} }}\left( {1 + \left( {1 - \phi } \right)^{2.5} We} \right)PrEc\left( {\begin{array}{*{20}c} {2F^{2} + G^{{\prime}{2}} + 2H^{{\prime}{2}} + 2\left( {\eta F^{\prime} + F} \right)^{2} } \\ { + \left( {F^{\prime} - \eta H^{\prime} - H} \right)^{2} + \left( {\eta G^{\prime} + 2G} \right)^{2} } \\ \end{array} } \right) \hfill \\ \end{gathered} $$

20 $$ \left( {1 + \eta^{2} } \right)\chi^{^{\prime\prime}} + \eta \chi^{\prime} + Sc\left( {\eta F - H} \right)\chi^{\prime} + \frac{Nt}{{Nb}}\left( {\left( {1 + \eta^{2} } \right)\theta^{\prime\prime} + \eta \theta^{\prime}} \right) - R_{c} Sc \chi = 0 $$

Conditions described in Eq. (12) are modified as below:

21 $$ \begin{array}{*{20}c} {F\left( 0 \right) = 1, G\left( 0 \right) = Re_{\omega } , H\left( 0 \right) = 0, \theta \left( 0 \right) = 1, \chi \left( 0 \right) = 1} \\ {F\left( {\eta_{0} } \right) = 0, G\left( {\eta_{0} } \right) = Re_{\Omega } , H\left( {\eta_{0} } \right) = 0, \theta \left( {\eta_{0} } \right) = 0, \chi \left( {\eta_{0} } \right) = 0} \\ \end{array} $$

where $${\eta }_{0}=tan\gamma .$$

## Physical quantities

The skin friction coefficients ${Cf}_{r}$ and ${Cf}_{\theta }$ across the $r$ and $\theta$ directions, as well as the Nusselt number $Nu$ and the Sherwood numbe r $Sh$, are the physical extents of concern in this consideration.

22 $$ Cf_{r} = \frac{{\left. {\tau_{r} } \right|_{z = 0} }}{{\rho_{f} U_{w}^{2} }} $$

23 $$ Cf_{\theta } = \frac{{\left. {\tau_{\theta } } \right|_{z = 0} }}{{\rho_{f} U_{w}^{2} }} $$

24 $$ Nu = \frac{{r\left. {q_{w} } \right|_{z = 0} }}{{k_{f} \left( {T_{w} - T_{\infty } } \right)}} $$

25 $$ Sh = \frac{{r\left. {q_{m} } \right|_{z = 0} }}{{D_{B} \left( {C_{w} - C_{\infty } } \right)}} $$

The boundary conditions described in Eqs. (22–25) have been modified as follows:

26 $$ Re_{\omega }^{2} Cf_{r} = \frac{1}{{\left( {1 - \phi } \right)^{2.5} }}\left( {1 + \left( {1 - \phi } \right)^{2.5} We} \right)F^{\prime}\left( 0 \right) $$

27 $$ Re_{\omega }^{2} Cf_{\theta } = \frac{1}{{\left( {1 - \phi } \right)^{2.5} }}\left( {1 + \left( {1 - \phi } \right)^{2.5} We} \right)G^{\prime}\left( 0 \right) $$

28 $$ Nu = - \frac{{k_{nf} }}{{k_{f} }}\left( {1 + \frac{{k_{f} }}{{k_{nf} }}Rd} \right)\theta^{\prime}\left( 0 \right) $$

29 $$ Sh = \chi^{\prime}\left( 0 \right) $$

The following provides an easy way to illustrate the problem's non-dimensional parameters:

30 $$ \begin{gathered} e = \frac{1}{{\mu_{f} Bc_{1} }}, \beta = \frac{{\mu_{f} r}}{{\rho_{f} KU_{w} }}, M = \frac{{\sigma_{f} B_{0}^{2} r}}{{\rho_{f} U_{w} }}, Rd = \frac{{16\sigma^{*} T_{\infty }^{3} }}{{3k^{*} k_{f} }}, Pr = \frac{{\upsilon_{f} \left( {\rho C_{p} } \right)_{f} }}{{k_{f} }}, Nt = \frac{{\tau D_{T} \left( {T_{w} - T_{\infty } } \right)}}{{\left( {\rho C_{p} } \right)_{f} \upsilon_{f} T_{\infty } }}, \hfill \\ Ec = \frac{{\upsilon_{f}^{2} }}{{r^{2} C_{p} \left( {T_{w} - T_{\infty } } \right)}}, Nb = \frac{{\tau D_{B} \left( {C_{w} - C_{\infty } } \right)}}{{\left( {\rho C_{p} } \right)_{f} \upsilon_{f} }}, Sc = \frac{{\upsilon_{f} }}{{D_{B} }}, R_{c} = \frac{{R_{1} r^{2} }}{{\upsilon_{f} }}, Re_{\omega } = \frac{{\omega r^{2} }}{{\upsilon_{f} }}, Re_{\Omega } = \frac{{\Omega r^{2} }}{{\upsilon_{f} }} \hfill \\ \end{gathered} $$

## Entropy generation and Bejan number

Any system's formation of entropy must be considered in ordert to recognize how thermal energy in the system is irreversible. The existing model's prime objective is to reduce entropy formation by influencing a range of physical features in order to achieve improved outcomes. The following mathematical formula can be utilized to conclude the current model's entropy production ratio per unit volume.

31 $$ \begin{aligned} S_{gen} &= \frac{1}{{T_{\infty }^{2} }}\left( {k_{nf} + \frac{{16\sigma^{*} T_{\infty }^{2} }}{{3k^{*} }}} \right)\left( {\left( {\frac{\partial T}{{\partial r}}} \right)^{2} + \left( {\frac{\partial T}{{\partial z}}} \right)^{2} } \right) + \frac{{RD_{B} }}{{C_{\infty } }}\left( {\left( {\frac{\partial T}{{\partial r}}} \right)^{2} + \left( {\frac{\partial T}{{\partial Z}}} \right)^{2} } \right) \hfill \\ &\quad+ \frac{{RD_{B} }}{{T_{\infty } }}\left( {\frac{\partial T}{{\partial Z}}\frac{\partial C}{{\partial Z}} + \frac{\partial T}{{\partial r}}\frac{\partial C}{{\partial r}}} \right) + \frac{{\sigma_{nf} B_{0}^{2} }}{{T_{\infty } }}\left( {u^{2} + v^{2} } \right)  \hfill \\&\quad+ \frac{1}{{T_{\infty } }}\left( {\mu_{nf} + \frac{1}{{Bc_{1} }}} \right)\left[ {2\left( {\frac{\partial u}{{\partial r}}} \right)^{2} + 2\left( {\frac{\partial w}{{\partial z}}} \right)^{2} + \left( {\frac{\partial v}{{\partial z}}} \right)^{2} + 2\left( \frac{u}{r} \right)^{2} + \left( {\frac{\partial u}{{\partial z}} + \frac{\partial w}{{\partial r}}} \right)^{2} + \left( {\frac{\partial v}{{\partial r}} - \frac{v}{r}} \right)^{2} } \right] \hfill \\ \end{aligned} $$

The following is the non-dimensional form of entropy production:

32 $$ N_{G} = \frac{{T_{\infty } r^{2} }}{{k_{f} \left( {T_{w} - T_{\infty } } \right)}}S_{gen} $$

The similarity transformation can be used to reduce the non-dimensional entropy formation to the following structure.

33 $$ \begin{aligned} N_{G} & = \underbrace {{\left( {\alpha_{1} - 1} \right)\frac{{k_{nf} }}{{k_{f} }}\left( {1 + \eta^{2} } \right)\left( {1 + \frac{{k_{f} }}{{k_{nf} }}Rd} \right)\theta^{{\prime}{2}} }}_{{N_{Gh} }} + \underbrace {{\frac{{\sigma_{nf} }}{{\sigma_{f} }}MBr\left( {F^{2} + G^{2} } \right)}}_{{N_{GJ} }} \hfill \\ &\quad+ \underbrace {{L\frac{{\left( {\alpha_{2} - 1} \right)^{2} }}{{\left( {\alpha_{1} - 1} \right)}}\left( {1 + \eta^{2} } \right)\chi^{{\prime}{2}} + L\left( {\alpha_{2} - 1} \right)\left( {1 + \eta^{2} } \right)\theta^{\prime}\chi^{\prime}}}_{{N_{Gm} }} \hfill \\ \&\quad+ \underbrace {{\frac{1}{{\left( {1 - \phi } \right)^{2.5} }}\left( {1 + \left( {1 - \phi } \right)^{2.5} We} \right)Br\left( {\begin{array}{*{20}c} {2F^{2} + G^{{\prime}{2}} + 2H^{{\prime}{2}} + 2\left( {\eta F^{\prime} + F} \right)^{2} } \\ { + \left( {F^{\prime} - \eta H^{\prime} - H} \right)^{2} + \left( {\eta G^{\prime} + 2G} \right)^{2} } \\ \end{array} } \right)}}_{{N_{GF} }} \hfill \\ \end{aligned} $$

The total entropy production of the system is denoted by ${N}_{G}$. ${N}_{Gh}$ is the entropy number resulting from thermal irreversibility. The entropy production number as a consequence of joule dissipation is defined by ${N}_{GJ}$. The entropy number due to irreversible mass transmission is denoted by ${N}_{Gm}$. The entropy number due to viscous dissipation is denoted by ${N}_{GF}$.

An essential factor in assessing entropy creation is the Bejan number. In light of this, the dimensionless Bejan number is constructed mathematically in the manner described below:

34 $$ Be = \frac{{N_{Gh} }}{{N_{G} }} = \frac{Entropy\; generation\; due \;to \;heat\; transfer}{{Total \;entropy \;generation}} $$

## Results and discussions

Under particular boundary conditions, the suggested mathematical model is used to numerically explore the effects of a porous medium, radiation, viscous and Joule dissipations, Brownian motion, thermophoresis, and chemical reaction. The present physical problem model consists of steady and incompressible nonlinear PDE’s, The shooting technique (bvp4c) with the necessary boundary conditions is used to solve a group of nonlinear ordinary differential equations that were produced using similarity transformations.It is researched and visually depicted how different values of the provided parameters infect the flow field. Included in this list are the Ree-Eyring fluid parameter, magnetic number, porosity parameter, Eckert number, chemical reaction parameter, Brownian motion, thermophoretic, Schmidt number, Hall recent, radiation, and volume fraction parameters.

### Discussion on tangential and radial velocity profile

**Figure 2:**
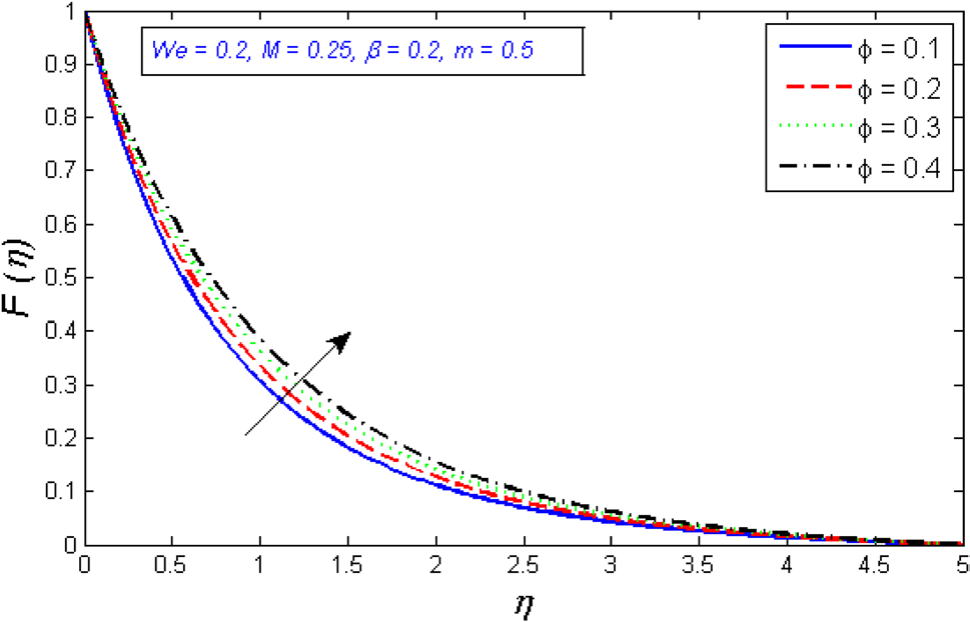
Represents variation of radial velocity distribution for several number of volume fraction $\phi$.

**Figure 3:**
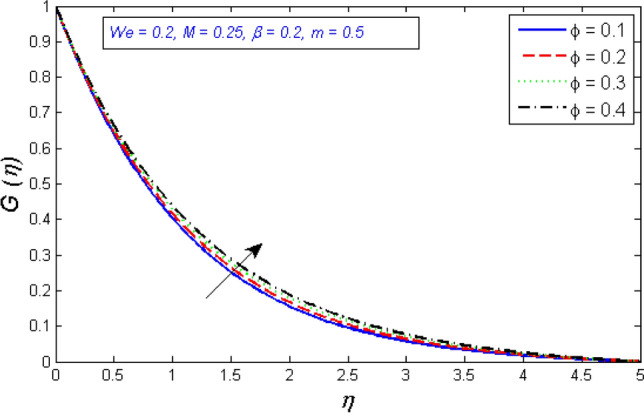
Represents variation of tangential velocity distribution for several number of volume fraction $\phi$.

The impact of volume fraction on the tangential and radial components of velocity is seen in Figs. 2 and 3. The redial and tangential velocity grows up as the volume fraction is enhanced. Because as rises, the fluid becomes less viscous, this accelerates the nanofluid velocity.

**Figure 4:**
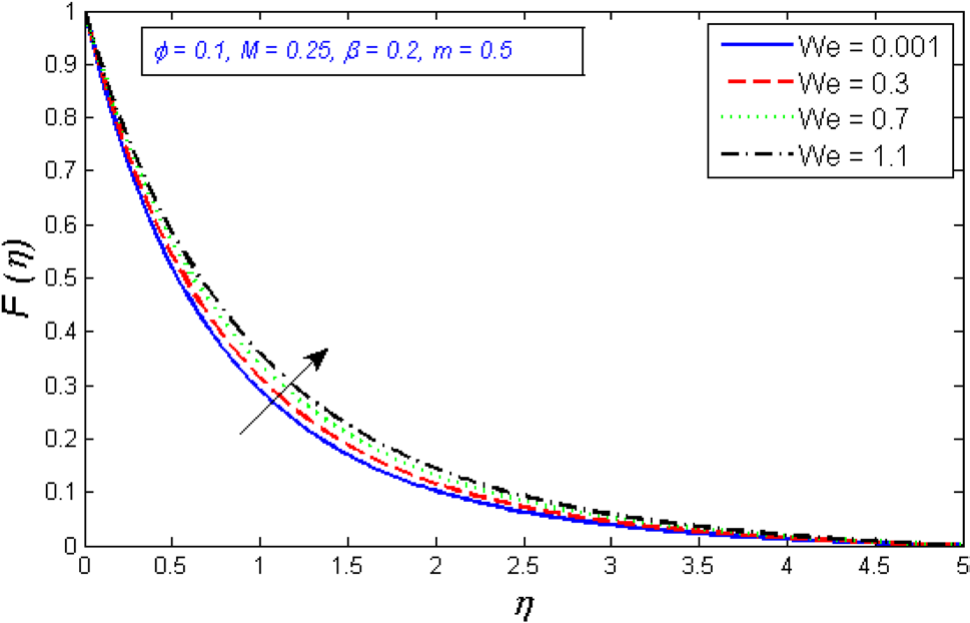
Variation of radial velocity distribution for several number of Ree-Eyring Weissenberg number $We$.

**Figure 5:**
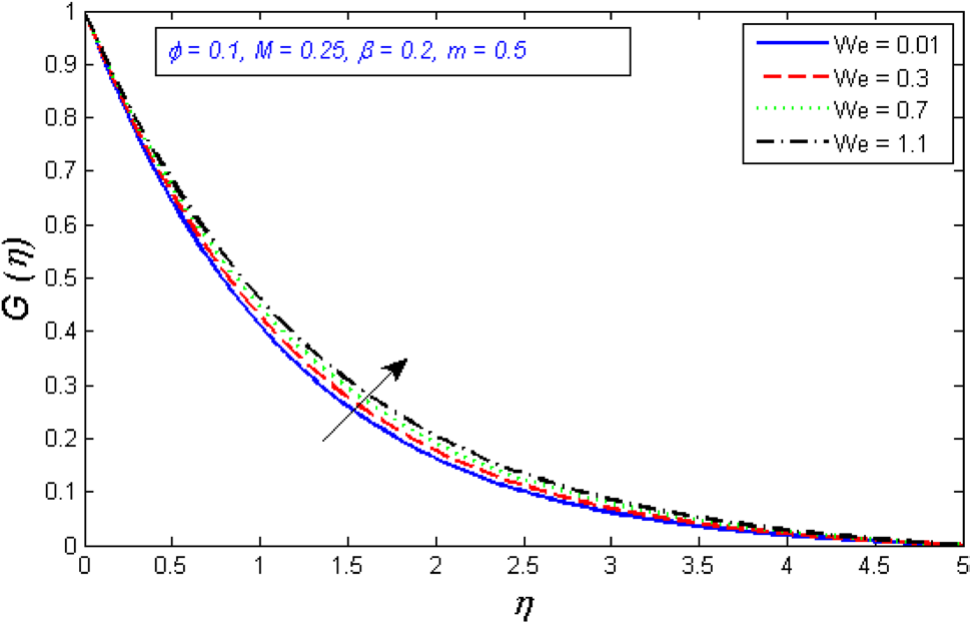
Represents variation of tangential velocity distribution for several number of Ree-Eyring Weissenberg number $We$.

Figures 4 and 5 show the results of the Weissenberg number $We$ focus on tangential velocity profiles and redial. The outcomes show that both velocity components are enhanced as the Weissenberg number $We$ boost up. Mathematically, Weissenberg number $We$ is applied to the study of viscoelastic flows. It is the difference between elastic and viscous forces. As a consequence, as the Weissenberg number grows, the viscous forces drop significantly and the velocity profile intensifies.

**Figure 6:**
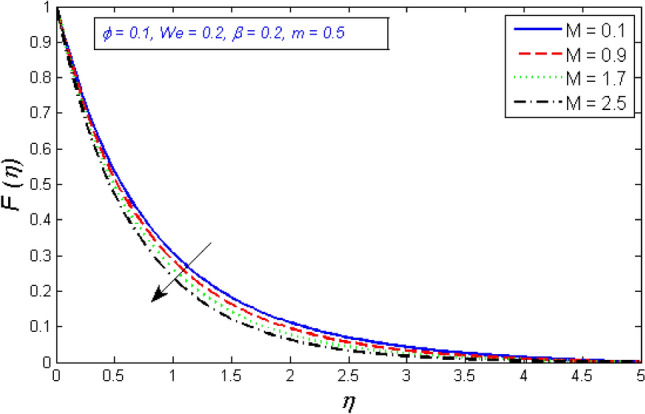
Represents variation of radial velocity distribution for several number of magnetic parameter $M$.

**Figure 7:**
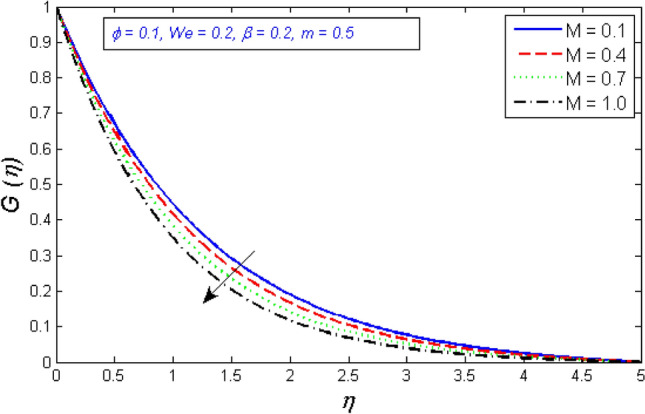
Variation of tangential velocity dispensation for several number of magnetic parameter $M$.

Figures 6 and 7 depict how the magnetic parameter M affects radial and tangential velocity. As the parameter M increases in strength, the radial and tangential velocity decreases. Fluid velocity drops because the Lorentz force physically blocks flow and becomes stronger as M rises.

**Figure 8:**
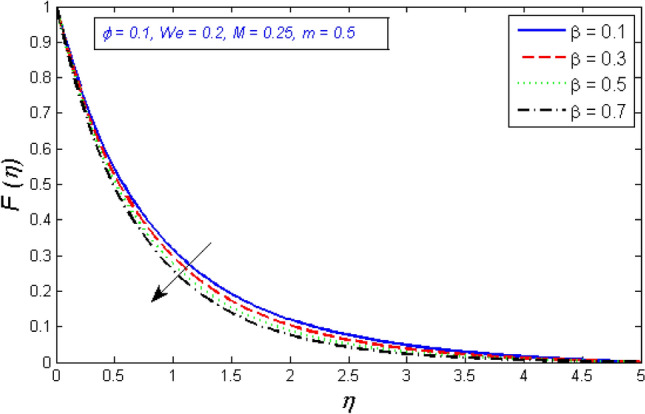
Represents variation of radial velocity distribution for several number of porosity parameter $\beta$.

**Figure 9:**
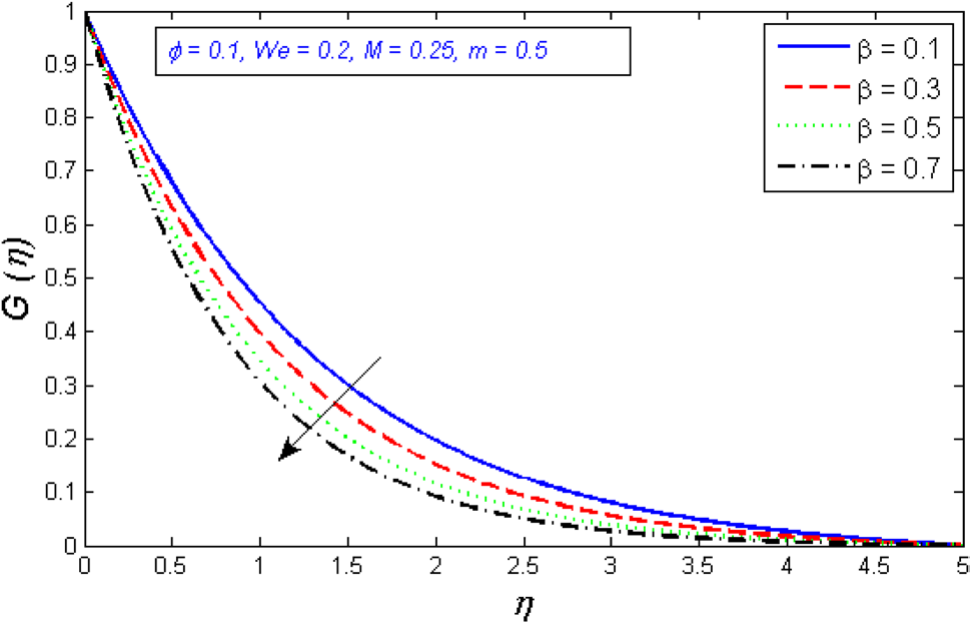
Represents variation of tangential velocity distribution for several number of porosity parameter $\beta$.

Figures 8 and 9 show the effects of the porosity parameter on the radial and tangential components of the velocity profile. The enhancement in the porous parameter of the fluid is because of an increment of the fluid viscosity or reduction in the permeability at the edge, this will result in a gradual decrease in the flow fluid viscosity, which is demonstrated in Figs. 8 and 9.

**Figure 10:**
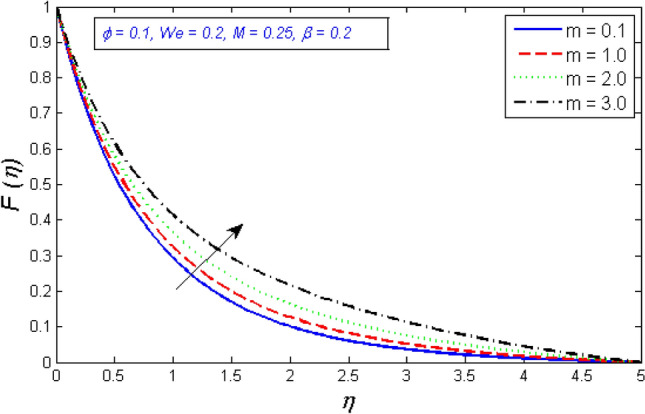
Represents variation of radial velocity distribution for several number of Hall parameter $m$.

**Figure 11:**
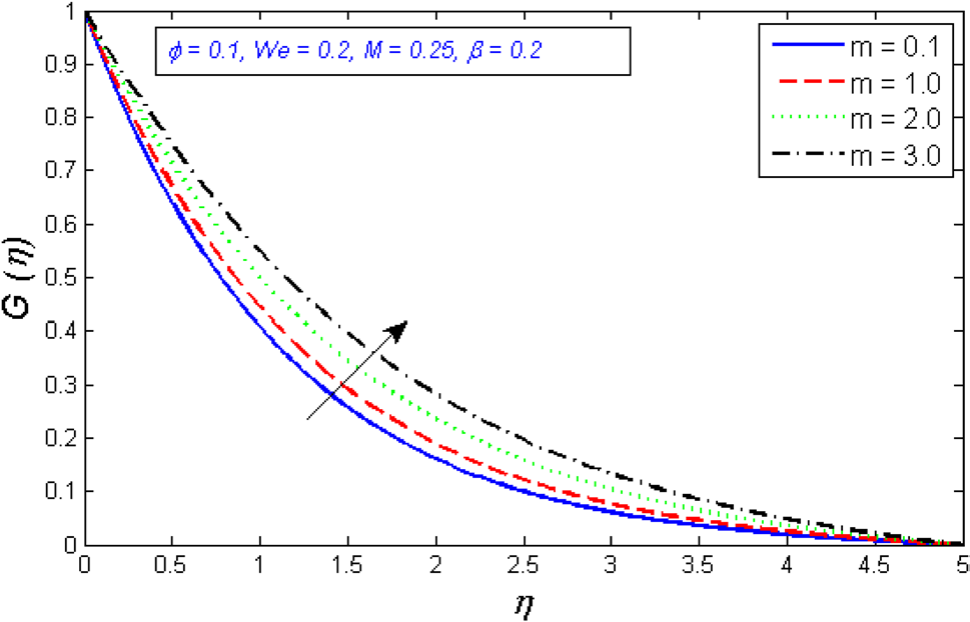
Represents variation of tangential velocity distribution for several number of Hall parameter $m$.

Figures 10 and 11 show how the velocity profile's radial and tangential components change as a function of the Hall parameter m. With the enhancement in the Hall parameter, the velocity components are enhanced. An increase in hall parameter $m$ indicates that either electron frequency or electron collision time is increased, or that both values are increased. It is also a proven fact that magnetic and Hall forces are diametrically opposed. As previously stated, magnetic forces resist fluid motion. According to the concept, the Hall Effect aids fluid velocity.

**Figure 12:**
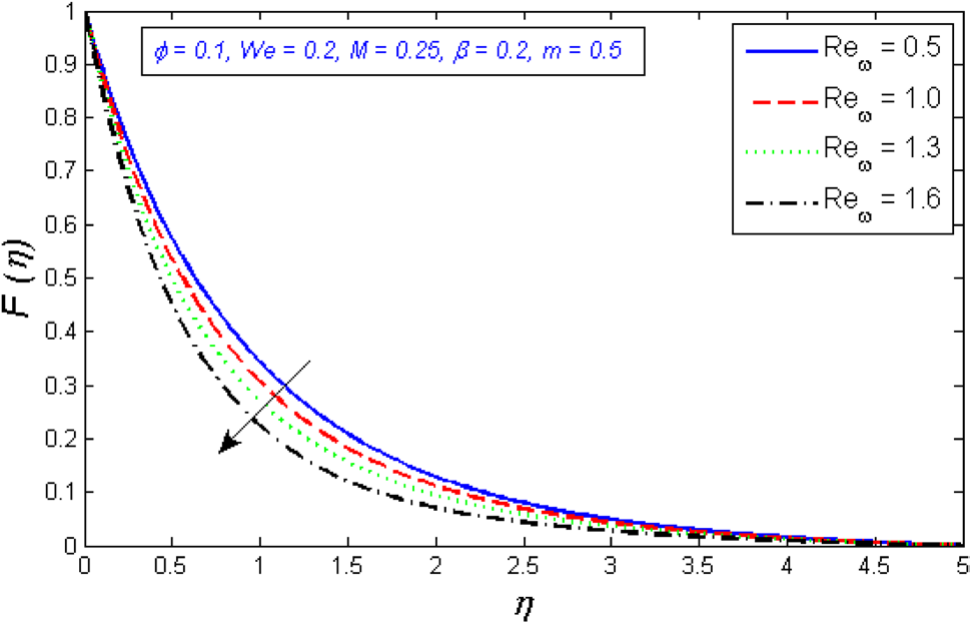
Represents variation of radial velocity distribution for several number of Local Reynolds number established on the disc angular velocity ${Re}_{\omega }$.

**Figure 13:**
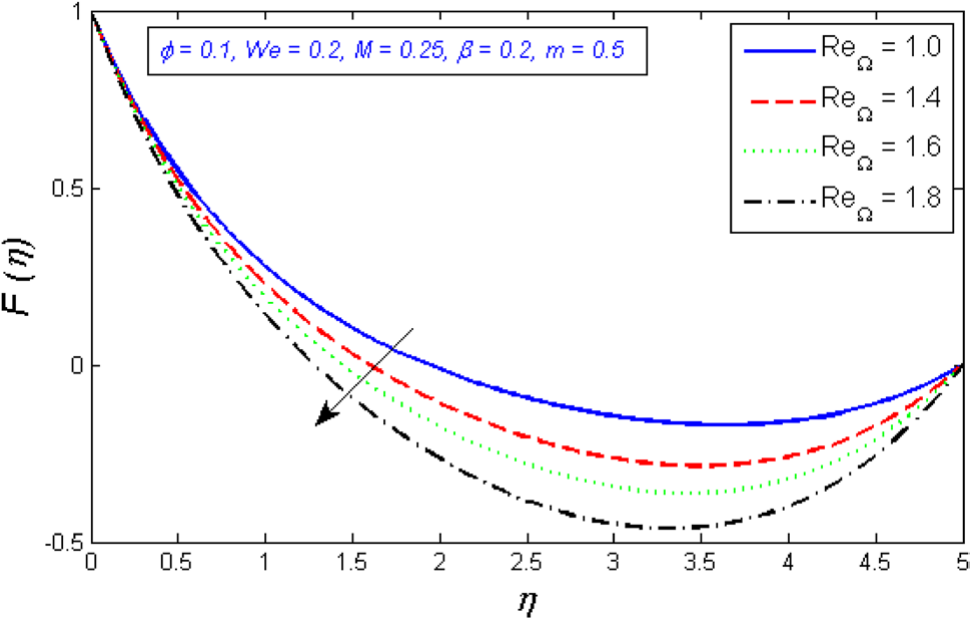
Represents variation of radial velocity distribution for several number of Local Reynolds number established on the cone angular velocity ${Re}_{\Omega }$.

Figure 12 and 13 depict the behavior of the radial velocity in relation to the impact of the two Reynolds numbers, ${Re}_{\omega }$&${Re}_{\Omega }$. It is discovered that as the two Reynolds numbers raise, the radial velocity significantly reduces; however, at the unbound stream, it vanishes. The Reynolds number is a physical illustration of the ratio of inertial and viscous forces that are due to the relative internal movement of different fluid velocities. More friction results from this relative movement, which lowers radial velocity.

**Figure 14:**
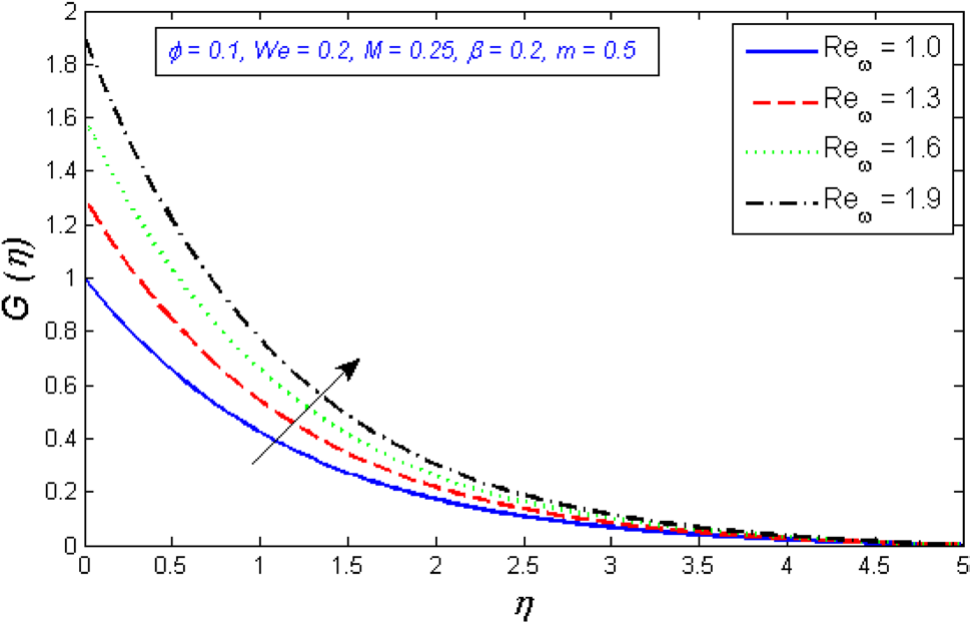
Variation of tangential velocity distribution for several number of Local Reynolds number established on the disc angular velocity ${Re}_{\omega }$.

**Figure 15:**
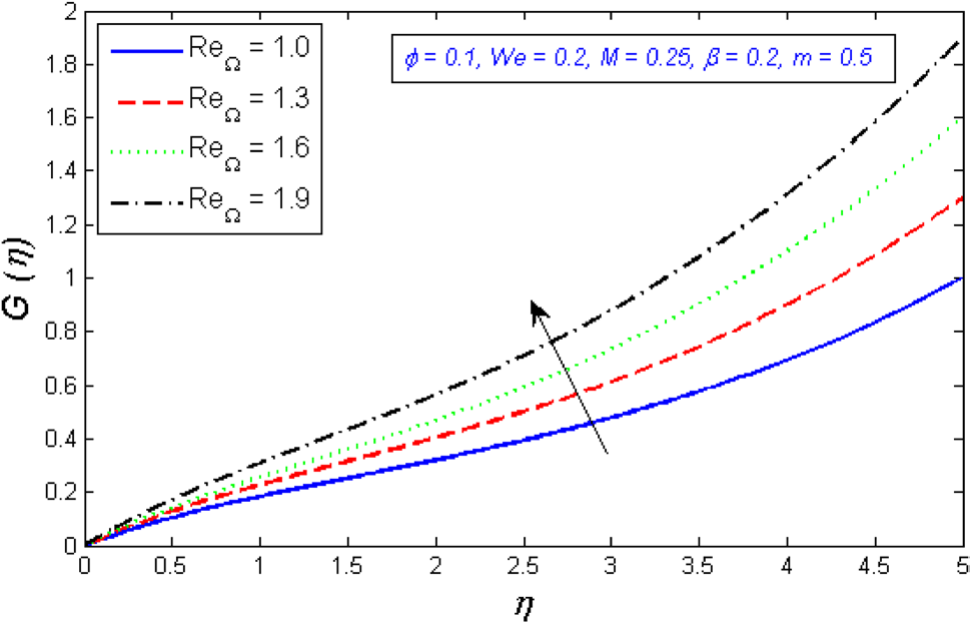
Variation of tangential velocity distribution for several number of Local Reynolds number established on the cone angular velocity ${Re}_{\Omega }$.

Figures 14 and 15 are intended to explain the behavior of the tangential velocity in relation to the impact of the two Reynolds numbers, ${Re}_{\omega }$&${Re}_{\Omega }$. These plots show that the tangential velocity rises as both ${Re}_{\omega }$ and ${Re}_{\Omega }$ increase. Because tangential velocity acts in the direction of rotation, increasing ${Re}_{\omega }$ and ${Re}_{\Omega }$ naturally increases tangential velocity.

### Discussion on temperature profile

**Figure 16:**
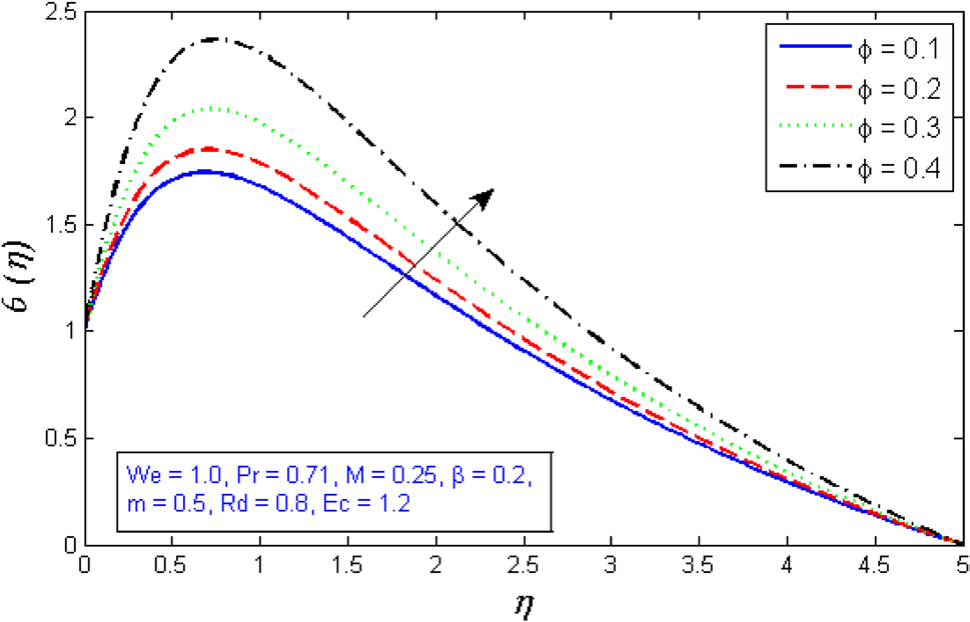
Represents variation of temperature distribution for several number of volume fraction $\phi$.

Figure 16 depicts the influence of volume fraction parameter $\phi$ on temperature profile. The figure shows that as the volume fraction rises, the temperature profile does as well. Because the augmented in volume fraction enhanced the thermal conductivity and therefore, falling the thickness of thermal boundary layer.

**Figure 17:**
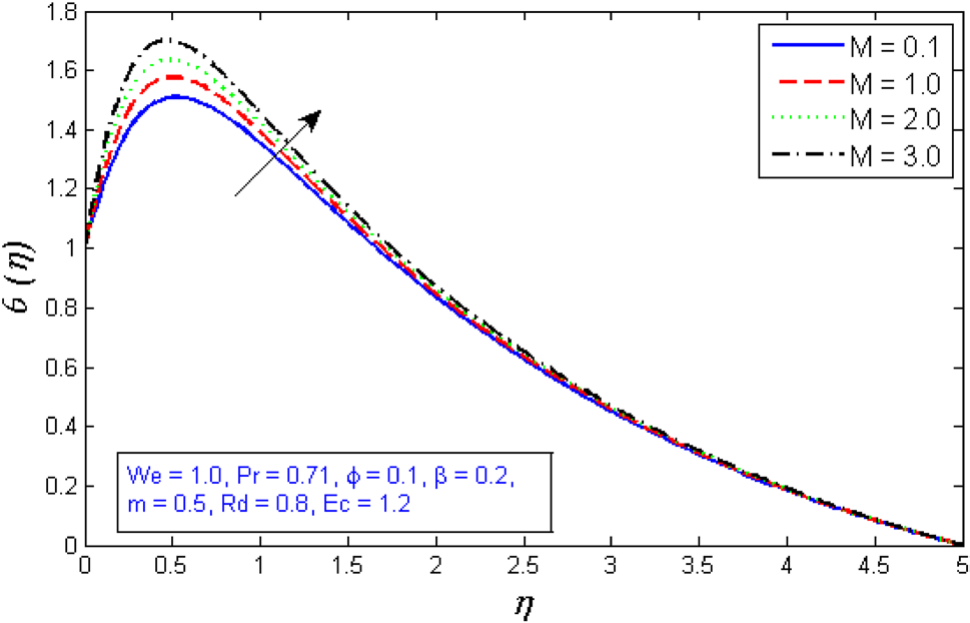
Variation of temperature distribution for several number of magnetic parameter $M$.

Figure 17 show the temperature rising as the magnetic parameter M rises. As mentioned before, increasing the magnetic field enhances the Lorentz force that opposes the flow, which increases thermal diffusion, as depicted in Fig. 17.

**Figure 18:**
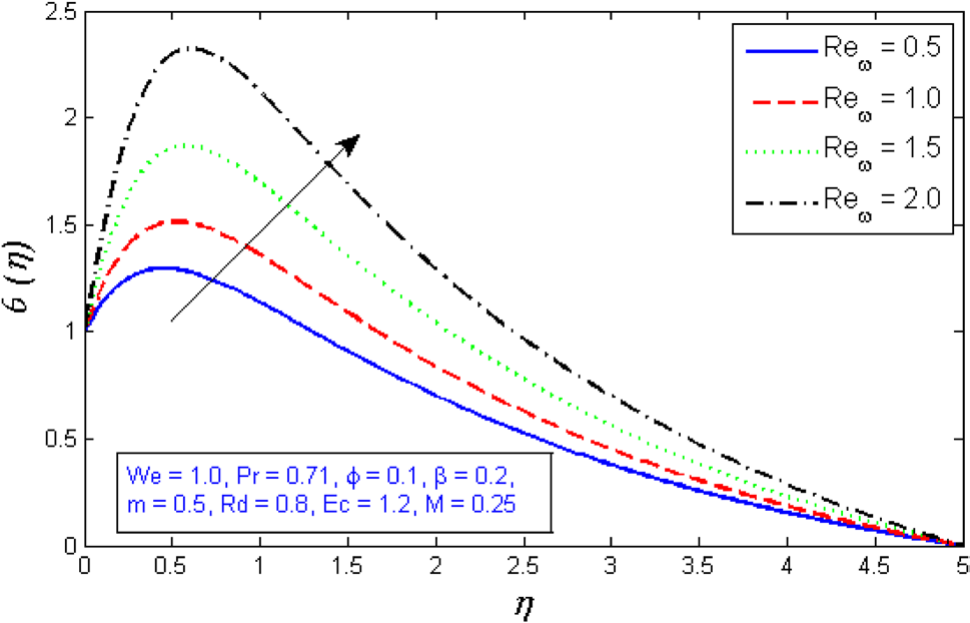
Represents variation of temperature distribution for numerous number of Local Reynolds number established on the disc angular velocity ${Re}_{\omega }$.

**Figure 19:**
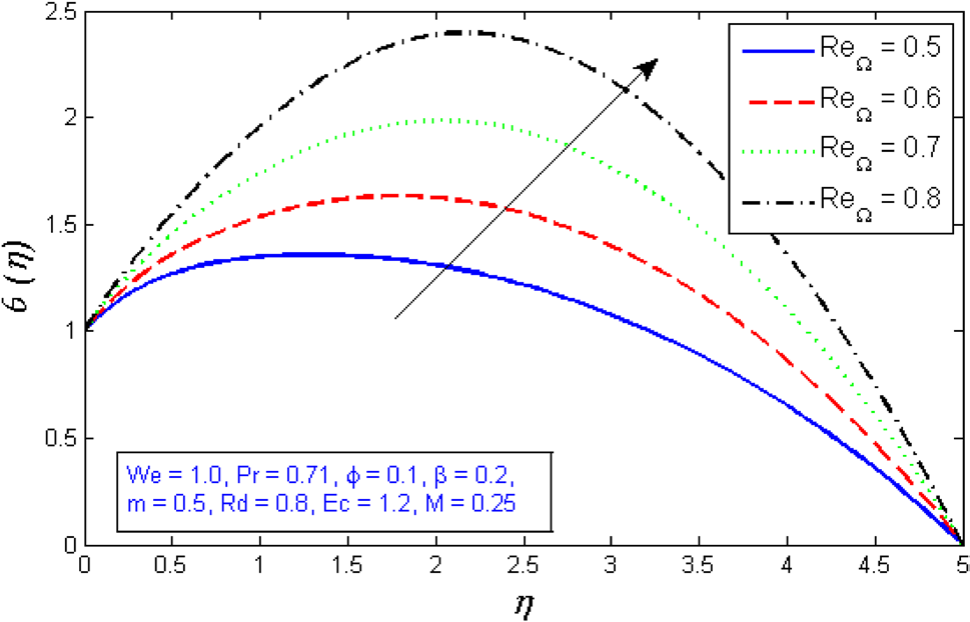
Represents variation of temperature distribution for several number of Local Reynolds number based on the disc angular velocity ${Re}_{\Omega }$.

The influences of the Reynolds numbers ${Re}_{\omega }$ and ${Re}_{\Omega }$ on the temperature profile are shown in Figs. 18 and 19. The temperature distribution declines as ${Re}_{\omega }$ and ${Re}_{\Omega }$ boost, as shown by these figures. As earlier noticed from the physical sense of ${Re}_{\omega }$ and ${Re}_{\Omega }$, greater values of these parameters result in a lower value of viscosity, and thus the temperature drops.

**Figure 20:**
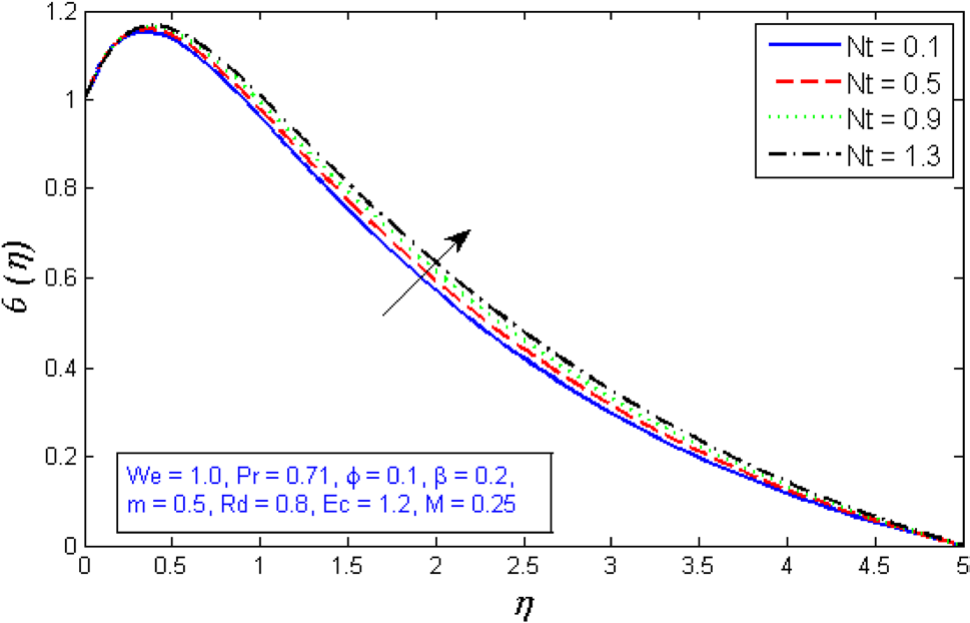
Represents variation of temperature distribution for several number of thermophoresis parameter $Nt$.

**Figure 21:**
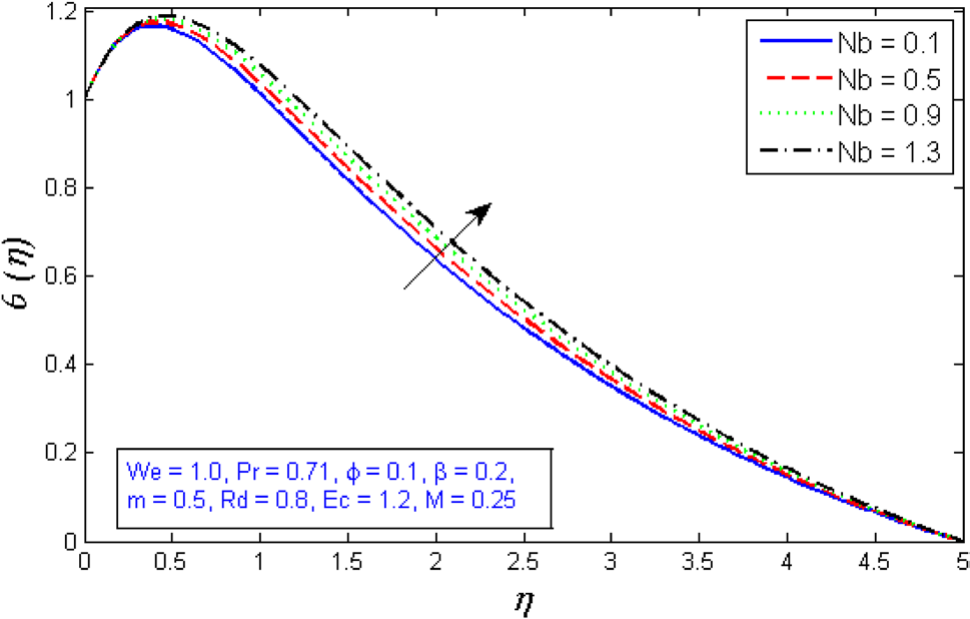
Represents variation of temperature distribution for several number of Brownian motion $Nb$.

The temperature curves for enhancing the thermophoresis parameter $Nt$ are depicted in Fig. 20. The proportion of momentum diffusion in nanofluids to diffusion of nanomaterials by thermophoresis force. The thermophoresis parameter is actually increased to raise the fluid temperature. Nanoparticles migrate from the warm to the cold sections of a temperature gradient. Therefore, the thickness of the thermal boundary sheet and the temperature distribution both rise as $Nt$ grows. The highlight of Brownian motion parameter $Nb$ on temperature field is shown in Fig. 21. As the Brownian motion parameter $Nb$ is improved, further heat is produced by the random collision of nanoparticles. As a consequence, the boundary film thickness and temperature field risen.

**Figure 22:**
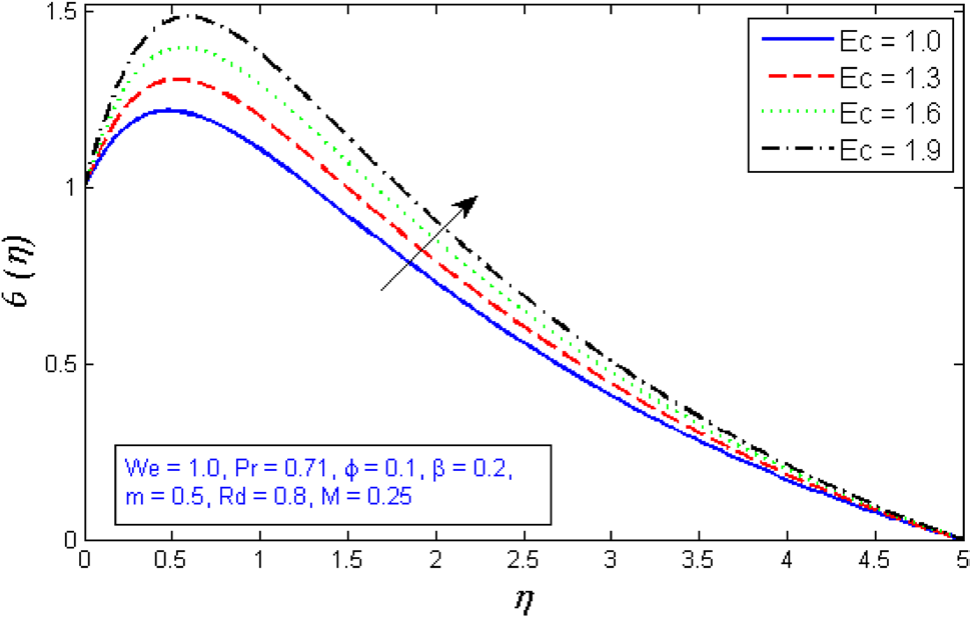
Represents variation of temperature distribution for numerous number of Eckert number $Ec$.

The Eckert number $Ec$ characterises heat transfer dissipation by expressing the association among the flow kinetic energy and the boundary film enthalpy transformation. As shown in Fig. 22, heat intemperance generates heat due to interaction between fluid particles, causing a rise in the new fluid temperature.

### Discussion on concentration profile

**Figure 23:**
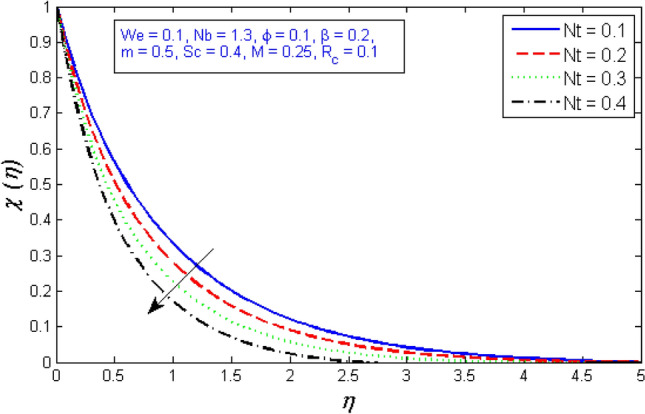
Represents variation of concentration profile for numerous number of thermophrosisparameter $Nt$.

**Figure 24:**
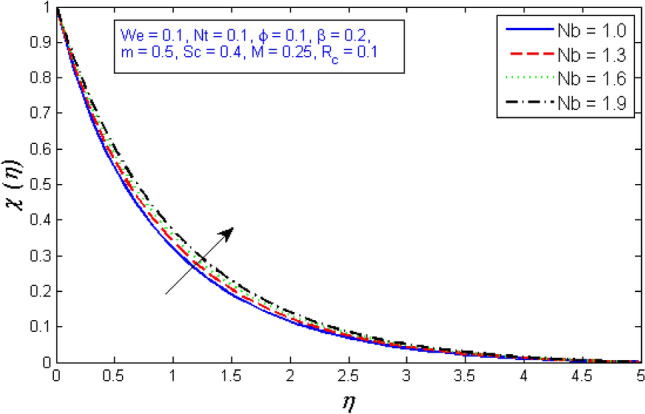
Represents variation of concentration profile for numerous number of Brownian motion $Nb$.

**Figure 25:**
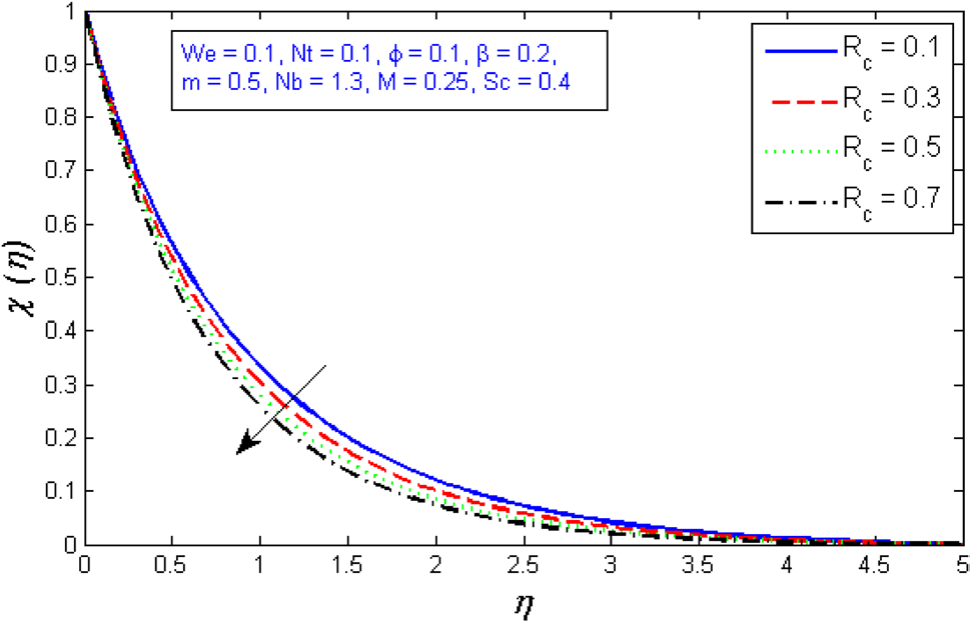
Represents variation of concentration profile for numerous number of Chemical reaction ${R}_{c}$.

**Figure 26:**
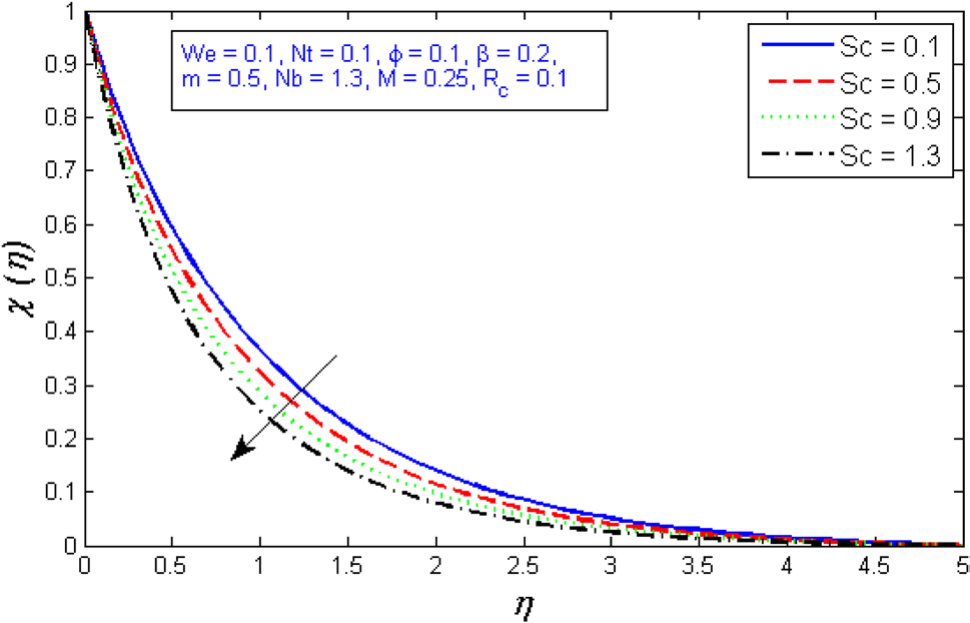
Represents variation of concentration profile for several number of Schmidt number $Sc$.

The impacts of the parameters: the thermophrosisparameter $Nt$, Brownian motion $Nb$, the chemical reaction parameter ${R}_{c}$, and the Schmidt number $Sc$, of the concentration distribution phi are plotted in Figs. 23, 24, 25and26, respectively. The boost in the parameter $Nt$ provides a logical and physical description for the decrement in $\chi$, with the nanoparticles dispersing and accelerating in their casual motion as shown in Fig. 23. Figure 24 specifies the impact of the Brownian motion parameter $Nb$ on the $\chi$ profile. The $\chi$ sharing is reported to boost as $Nb$ enhances. Brownian motion is a casual association of particles suspension in a fluid. This random motion enhances as $Nb$ increases, implying greater nanoparticle divergence. According to Fig. 25, the concentration of nanoparticles $\chi$ reduces as the chemical reaction parameter ${R}_{c}$ enhances. Physically, as ${R}_{c}$ improves, mass diffuses rapidly through the surrounding fluid. As a result, it scatters nanoparticles away from the flow, causing in a reduction in nanoparticle concentration $\chi$. As illustrated in Fig. 26, the concentration distribution $\chi$ significantly reduces as the Schmidt number $Sc$ grows. According to the certainty that the Schmidt number signifies the ratio of momentum to mass diffusivities, the mass diffusivity reduces as $Sc$ rises, implying a reduction in $\chi$.

### Discussion on Entropy profile and Bejan number

**Figure 27:**
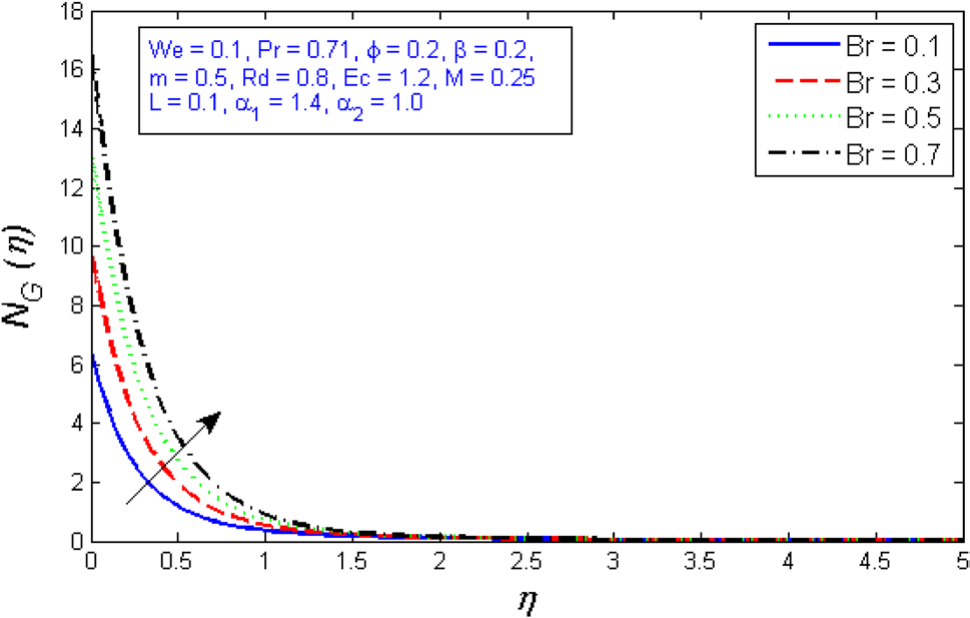
Represents variation of Entropy generation for several number of Brinkman number $Br$.

**Figure 28:**
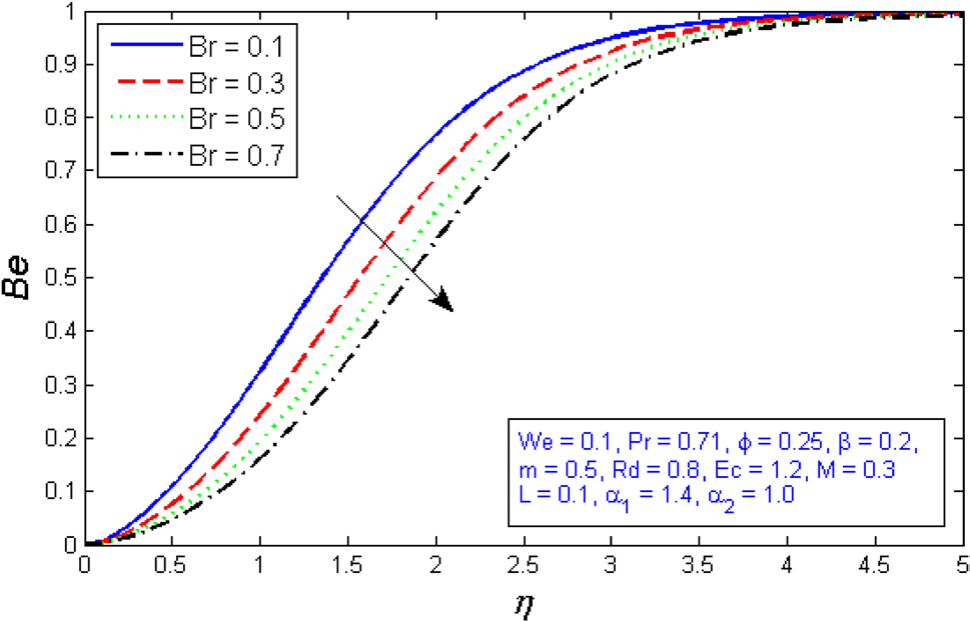
Represents variation of Bejan number for several number of Brinkman number $Br$.

Figures 27 and 28 depict the outcome of the Brinkman number $Br$ on the entropy production rate and Bejan number. The Brinkman number $Br$ compares the significance of heat created by viscous heating to heat held by molecular transmission. As the Brinkman number is improved, the entropy production number significantly increases, while Bejan number decreases.

**Figure 29:**
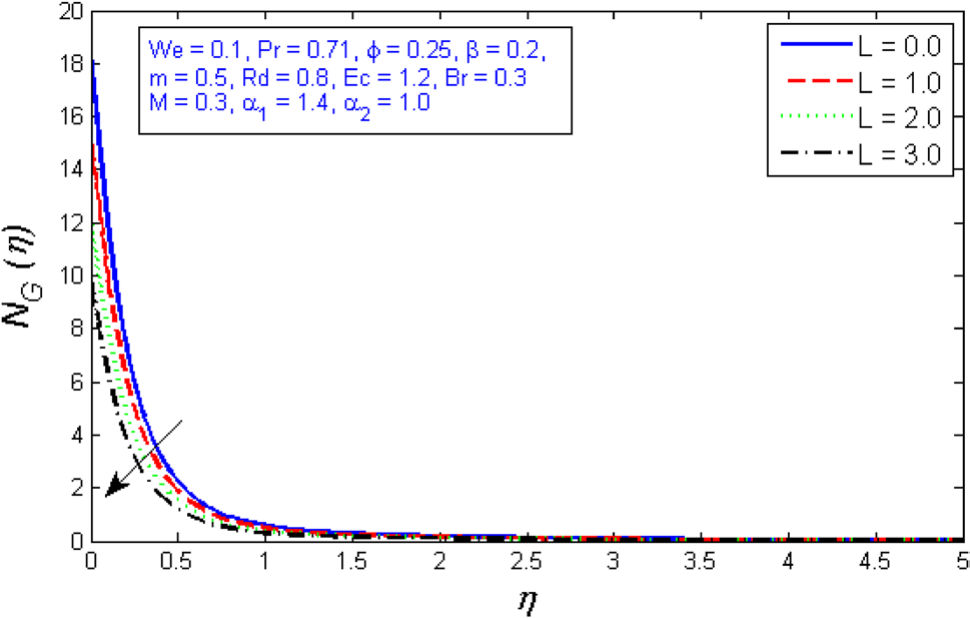
Represents variation of Entropy generation for several number of diffusion parameter $L$.

**Figure 30:**
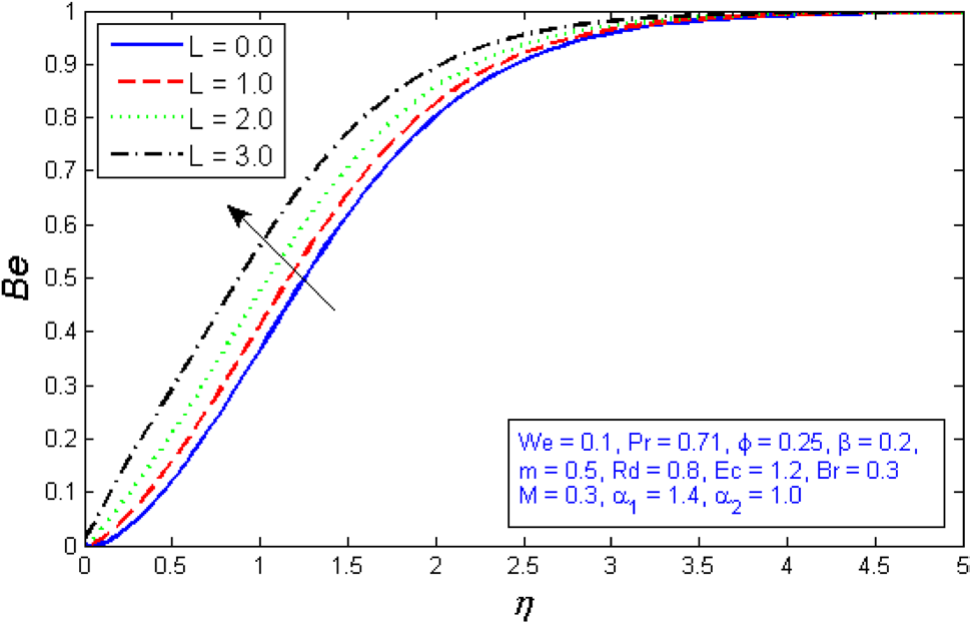
Represents variation of Bejan number for several number of diffusion parameter $L$.

Figures 29 and 30 are plotted against the entropy formation and Bejan number for several values of diffusion parameter $L$. The graphical results show that the Bejan number increases while the entropy generation rate decreases for large values of the diffusion parameter $L$.

**Figure 31:**
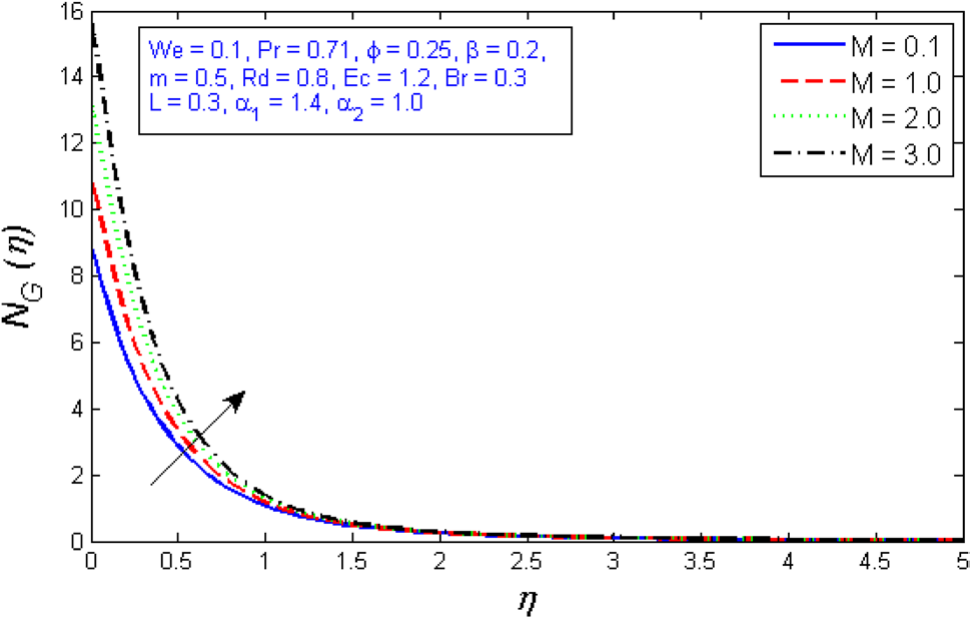
Variation of Entropy generation for several number of magnetic parameter $M$.

**Figure 32:**
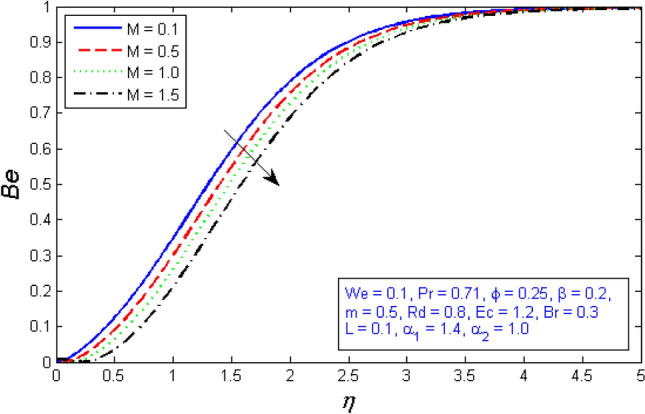
Represents variation of Bejan number for several number of magnetic parameter $M$.

Figures 31 and 32 show how the magnetic parameter $M$ affects entropy production and Bejan number. The graph shows that as the magnetic parameter increased, so did the entropy creation, whereas, the Bejan number decrease. Because the presence of magnetic flies generates more entropy in the fluid, the temperature of the fluid rises, and thus entropy production rises, while Bejan number declines.

## Discussion on Skin Friction and Nusselt number

Tables 2, 3and4 indicate the variation of different parameter on skin friction coefficient, Nusselt number, and Sherwood number. Table 2 depicts the disparity in skin friction coefficient in radial and tangential direction for different numbers of $\phi , M, We,$ and $\beta$. It is observed that the for bigger values of volume fraction $\phi$ and porosity parameter $\beta$, both the radial and tangential skin friction coefficient drop down. The radial skin friction coefficient decline as the magnetic and Weissenberg number rise up, while the tangential skin friction coefficient grow up for the same case. Table 3 demonstrates the change in Nusselt number $Nu$ for various values of $\phi , M, We$, and $Ec$. This table indicates that Nusselt number declined for huge values of volume fraction, Eckert number, magnetic, and Weissenberg number. Table 4 illustrates the change in Sherwood number for numerous values of $Sc, Nt,$ and $Nb$. The Table 4 indicates that when the Schmidt number and Brownian motion rise up the Sherwood number improves; however, the Sherwood number declined for higher thermophrosis parameter. Table 5 presents the validation of the results. The comparison values of Nusselt number with previous results are given. An excellent agreement is observed.

**Table 2:** Variation of different parameter on skin friction coefficient.

$\phi$	$M$	$\beta$	$We$	${Re}^{2}{Cf}_{r}$	${Re}^{2}{Cf}_{\theta }$
0.1	0.3	0.5	0.5	− 2.8463726	− 2.342097
0.2				− 3.3346007	− 2.8651318
0.3				− 4.2201747	− 3.7727065
0.1	0.1	0.5	0.5	− 2.7884657	− 2.2617008
	0.2			− 2.8175919	− 2.3024653
	0.3			− 2.8463726	− 2.342097
0.1	0.1	0.1	0.5	− 2.5232108	− 1.9988114
		0.2		− 2.5936896	− 2.069733
		0.3		− 2.661165	− 2.1368586
0.1	0.1	0.1	0.1	− 2.1438021	− 1.6293632
			0.3	− 2.3329559	− 1.8140702
			0.5	− 2.5232108	− 1.9988114

**Table 3:** Variation of different parameter on Nusset number.

$\phi$	$M$	$We$	$Ec$	$Nu$
0.1	0.1	0.5	0.2	− 1.6640069
0.2				− 2.1155872
0.3				− 2.8299755
	0.1		0.2	− 1.6640069
	0.2			− 1.6808579
	0.3			− 1.6984275
0.1	0.1	0.1	0.2	− 1.2435745
		0.3		− 1.4540545
		0.5		− 1.6640069
0.1	0.1	0.3	0.1	0.59575759
			0.2	− 1.4540545
			0.3	− 2.2994401

**Table 4:** Variation of different parameter on Sherwood number.

$Sc$	$Nt$	$Nb$	$Sh$
0.1	0.1	0.5	− 1.0052272
0.2			− 1.0469643
0.3			− 1.1135642
0.1	0.1		− 1.0052272
	0.2		− 1.2794194
	0.3		− 1.5544015
0.1	0.1	0.1	− 1.9980719
		0.2	− 1.3814679
		0.3	− 1.1740759

**Table 5:** Comparison values of Nusselt number with Literature^27^.

$M$	Present paper	Literature Moatimid et al.^27^
0	1.1175289	1.1175
0.1	1.1103288	1.114
0.2	1.1021636	1.1105
0.3	1.0928879	1.107
0.4	1.0823038	1.1035
0.5	1.0702154	1.1

## Statistical paradigm

The relationship coefficient is a statistical extent of the degree of relationship of two variables' comparative movements. Certain experiments in laboratory settings are difficult to perform when conducting various studies; in this instance, correlation studies are conducted. Wheezing attacks seem to be correlated with foggy days. Correlation statistics are also useful in finance and investing. Correlation, on the other hand, is used in statistics to demonstrate the relationship between two quantitative variables. We also assume the relationship is linear, with one factor changing by a fixed amount with every unit change by the other. The coefficient of correlation, *r, *indicates the strength of an association. A value between − 1 and 1 is considered. A designed number more than 1.0 or smaller than − 1.0 specifies that the association measurement was improper. A correlation of − 1.0 specifies a perfect negative correlation, while a correlation of 1.0 specifies a perfect positive correlation. A correlation of 0.0 specifies that there is no direct link between the movements of the two variables.

In this section, we broaden the focus of our examination to include the many implications of relevant parameter values on the topic under study. The correlation coefficient is calculated in order to improved understand the relationship between various physical parameters and the coefficient of skin friction, Sherwood number, and Nusselt number. We listed the numerical values for skin friction, Serwood number and the Nusselt number in Tables 2, 3 and 4, respectively, along with numerous relevant parameters of interest.

Following are some possible meanings for the correlation coefficient:

- When r = 1, there is an exact positive linear relationship between the two variables.
- If r = − 1, the connection between the two quantities is perfectly negative linear.
- A strong positive linear relationship between two quantities, indicated by $0.7 \le r \le 1.$
- Strongly negative linear relationship between quantities is shown by $- 1 \le r \le - 0.7.$
- r = 0, shows that the variables do not relate to one another linearly.

Table 6 displays a significant positive association between all physical parameters and skin friction coefficient while an exact positive linear relationship is noted for Wessenberg number We. Furthermore, if we discussed the linear correlation between physical attributes and Nusselt number, we discovered that while there is a strong positive association for all considered parameters. Moreover, according to local Sherwood number, there is a strong positive relationship for the all considered parameters except the Brownian motion parameter Nb.

**Table 6:** Numerical values of correlation coefficient for Skin friction and Nusselt number and Sherwood number.

Skin friction			
$r$	$- C_{f} Re_{x}^{{{1 \mathord{\left/ {\vphantom {1 2}} \right. \kern-\nulldelimiterspace} 2}}}$	$r$	${-Re}^{2}{Cf}_{r}$
$\phi$	0.9863426	$\phi$	0.9881715
$M$	0.9999941	$M$	0.8697447
$\beta$	0.9999210	$\beta$	0.8730234
$We$	0.9999986	$We$	1.0000000

Nusselt number		Sherwood number	
$r$	*−Nu*	$r$	*−Sh*
$\phi$	0.9916386	$Sc$	0.9913359
$M$	0.9999274	$Nt$	0.9999997
$We$	0.9999997	$Nb$	− 0.9612674
$Ec$	0.9999904		

### Probable error

Probable Error is primarily the correlation coefficient, which is exactly trustworthy for the coefficients' significance and exactness. It aids in determining the coefficient's reliability. The probable error is additional to or deducted from the correlation coefficient to determine the higher and lesser bounds within which the correlation coefficient value is meant to fall. The following formula can be used to calculate the Probable Error of Correlation Coefficient:

$$ P.E\left( r \right) = 0.6745 \times \frac{{\left( {1 - r^{2} } \right)}}{{\sqrt {\tilde{n}} }}, $$

where the number of observations is exemplified by “$\tilde{n}$” and “*r*” is the correlation coefficient. When the value of “r” is less than P.E., there is no correlation between the variables. TThis implies that the correlation coefficient is entirely insignificant. The correlation is considered certain when the value of “*r*” is six times greater than the feasible error; this indicates that the value of “*r*” is significant. P.E. is calculated to test the validity of the correlation coefficient value. Table 7 displays the likely error values for the Skin friction sherwood number and Nusselt number.

**Table 7:** Numerical values of P.E. for Skin friction and Nusselt number $NuRe_{x}^{{ - {1 \mathord{\left/ {\vphantom {1 2}} \right. \kern-\nulldelimiterspace} 2}}}$.

Skin friction			
$P.E.\left( r \right)$	$- C_{f} Re_{x}^{{{1 \mathord{\left/ {\vphantom {1 2}} \right. \kern-\nulldelimiterspace} 2}}}$	$P.E.\left( r \right)$	${-Re}^{2}{Cf}_{r}$
$\phi$	1.056440 × 10^–2^	$\phi$	9.158099 × 10^–3^
$M$	4.620941 × 10^–6^	$M$	9.484162 × 10^–2^
$\beta$	6.151598 × 10^–5^	$\beta$	9.261645 × 10^–2^
$We$	1.093296 × 10^–6^	$We$	1.112359 × 10^–9^

Nusselt number		Sherwood number	
$P.E.\left( r \right)$	*−Nu*	$P.E.\left( r \right)$	*−Sh*
$\phi$	6.485006 × 10^–3^	$Sc$	6.718746 × 10^–3^
$M$	5.656848 × 10^–5^	$Nt$	2.685490 × 10^–7^
$We$	2.044170 × 10^–7^	$Nb$	2.958251 × 10^–2^
$Ec$	7.455127 × 10^–6^		

### Statistical proclamation

The values of $\tfrac{r}{P.E.\left( r \right)}$ are listed in Table 8. Table 8 demonstrates that all values of the parameters investigated for t kin friction coefficient agree with the relationship $\tfrac{r}{P.E.\left( r \right)} > 6$. As a consequence, these variables are statistically significant. It is also perceived that according to local Nusselt number all variables are statistically significant. Furthermore local Sherwood number, the values of the parameter $Sc$ and $Nt$ are statistically significant as they satisfy the relation $\tfrac{r}{P.E.\left( r \right)} > 6$. While it is statistically insignificant for the parameter Nb as the above said relation is not satisfied.

**Table 8:** Numerical values of $\tfrac{r}{P.E.\left( r \right)}$ for Skin friction and Nusseltnumber $NuRe_{x}^{{ - {1 \mathord{\left/ {\vphantom {1 2}} \right. \kern-\nulldelimiterspace} 2}}}$.

Skin friction			
$\tfrac{r}{P.E.\left( r \right)}$	$- C_{f} Re_{x}^{{{1 \mathord{\left/ {\vphantom {1 2}} \right. \kern-\nulldelimiterspace} 2}}}$	$\tfrac{r}{P.E.\left( r \right)}$	${-Re}^{2}{Cf}_{r}$
$\phi$	93.36477	$\phi$	107.9014
$M$	216,404.9	$M$	9.170496
$\beta$	16,254.65	$\beta$	9.426224
$We$	9,146,640	$We$	898,989,985

Nusselt number		Sherwood number	
$\tfrac{r}{P.E.\left( r \right)}$	−*Nu*	$\tfrac{r}{P.E.\left( r \right)}$	−*Sh*
$\phi$	152.9125	$Sc$	147.5478
$M$	17,676.4	$Nt$	3,723,714
$We$	4,891,959	$Nb$	− 2.49445
$Ec$	134,134.6		

## Conclusions

The current study revisits real-world applications, primarily disk-cone apparatus used in industrial settings. A special form of Ree-Eyring nanoliquid containing copper Cu nanoparticles is being considered. Which are assumed to be moving or stationary in the occurrence of a magnetic field and porous medium. This study investigates the outcome of copper nanoparticles on the thermophysical characteristics of water. It has numerous uses in both science and technology. The flow equations are transformed into regular systems, and the shooting scheme is used to handle them (bvp4c). Figures and tables illustrate the effects of physical important variables on velocity, temperature, concentration, entropy production, and Bejan number. The following are the major conclusions:

- The radial and tangential components of velocity grow up for large number of volume fraction, Weissenberg number, and Hall parameters while drop down for higher number of magnetic and porosity parameter.
- When the of local Reynolds number established on the disc and cone angular velocity rise up, the radial velocity component fall down, whereas, the tangential velocity component grows up.
- The temperature distribution improves for improving the values of volume fraction, magnetic parameter, local Reynolds number based on the disc and cone angular velocity, thermophrosis, Brownian motion, and Eckert number.
- When the thermophrosis parameter, Chemical reaction, and Schmidt number upsurge, the concentration profile fall down, while when the Brownian motion parameter grows up the concentration profile rise up.
- The entropy production rises up for rising values of Brinkman number and magnetic parameter while falls down for greater value of diffusion parameter.
- The Bejan number fall down for higher values of Brinkman number and magnetic parameter, while grow up for large number of diffusion parameter. It is noticed that the Bejan number and entropy formation show contrasting behavior.
- It is observed that the for greater values of volume fraction $\phi$ and porosity parameter $\beta$, both the radial and tangential skin friction coefficient drop down.
- The radial skin friction coefficient decline as the magnetic and Weissenberg number rise up, while the tangential skin friction coefficient grow up for the same case.
- The Nusselt number declined for huge values of volume fraction, Eckert number, magnetic, and Weissenberg number.
- The Schmidt number and Brownian motion rise up the Sherwood number improves; however, the Sherwood number declined for higher thermophrosis parameter.

## Data Availability

The datasets used and/or analysed during the current study available from the corresponding author on reasonable request.
